# Gene regulatory effects of a large chromosomal inversion in highland maize

**DOI:** 10.1371/journal.pgen.1009213

**Published:** 2020-12-03

**Authors:** Taylor Crow, James Ta, Saghi Nojoomi, M. Rocío Aguilar-Rangel, Jorge Vladimir Torres Rodríguez, Daniel Gates, Rubén Rellán-Álvarez, Ruairidh Sawers, Daniel Runcie

**Affiliations:** 1 Department of Plant Sciences/University of California, Davis, California, United States of America; 2 Laboratorio Nacional de Genómica para la Biodiversidad/Unidad de Genómica Avanzada, Centro de Investigación y Estudios Avanzados (CINVESTAV- IPN), Irapuato CP 36821, Guanajuato, Mexico; 3 Corteva Agriscience, Agriculture Division of DowDuPont, Tlajomulco, Jalisco, Mexico; 4 Department of Evolution and Ecology/University of California, Davis, California, United States of America; 5 Department of Molecular and Structural Biochemistry, North Carolina State University, Raleigh, North Carolina, United States of America; 6 Department of Plant Science, Pennsylvania State University, State College, Pennsylvania, United States of America; University of Minnesota, UNITED STATES

## Abstract

Chromosomal inversions play an important role in local adaptation. Inversions can capture multiple locally adaptive functional variants in a linked block by repressing recombination. However, this recombination suppression makes it difficult to identify the genetic mechanisms underlying an inversion’s role in adaptation. In this study, we used large-scale transcriptomic data to dissect the functional importance of a 13 Mb inversion locus (*Inv4m*) found almost exclusively in highland populations of maize (*Zea mays* ssp. *mays*). *Inv4m* was introgressed into highland maize from the wild relative *Zea mays* ssp. *mexicana*, also present in the highlands of Mexico, and is thought to be important for the adaptation of these populations to cultivation in highland environments. However, the specific genetic variants and traits that underlie this adaptation are not known. We created two families segregating for the standard and inverted haplotypes of *Inv4m* in a common genetic background and measured gene expression effects associated with the inversion across 9 tissues in two experimental conditions. With these data, we quantified both the global transcriptomic effects of the highland *Inv4m* haplotype, and the local cis-regulatory variation present within the locus. We found diverse physiological effects of *Inv4m* across the 9 tissues, including a strong effect on the expression of genes involved in photosynthesis and chloroplast physiology. Although we could not confidently identify the causal alleles within *Inv4m*, this research accelerates progress towards understanding this inversion and will guide future research on these important genomic features.

## Author summary

Chromosomal inversions are a type of structural variant commonly found to underlie local adaptation and speciation. However, in most cases we do not know what genes or genetic variants inside an inversion are important, and how any such variants regulate the associated adaptive traits. Fine-mapping genetic variants using forward genetics inside inversions is difficult because recombination between heterokayotypes are suppressed. In this study, we used gene expression as a tool to study the genetics of a large chromosomal inversion found in highland maize populations in Mexico called *Inv4m*. Gene expression analyses in 9 tissues across two environmental conditions identified a large number of seemingly independent developmental and physiological processes regulated by *Inv4m*, suggesting that this locus contributes to local adaptation of maize to highland environments in multiple ways.

## Introduction

Chromosomal inversions are structural rearrangements that form when a portion of a chromosome breaks in two places and reinserts in the opposite orientation. The reversed order of loci inside an inversion greatly reduces recombination in heterozygous individuals because the crossover products are imbalanced in gene content and often non-viable [1]. This newly-formed genetic linkage can be important for speciation and local adaptation when haplotypes with alternative orientations carry multiple adaptive and potentially interacting loci in a linked block [2–4]. Inversions are common across taxa [1] and have been linked to adaptive phenotypes and environmental clines [5–7], mating system evolution [8–10], social organization [11], and migratory phenotypes [12], and can spread among populations through gene flow [13, 14].

Chromosomal inversions were first discovered nearly a century ago in *Drosophila* [3, 15] by visualizing karyotypes, and can also be identified based on their effects on recombination rates among nearby markers. Both techniques are labor intensive and difficult to apply to large-scale population-level surveys within or among species. However, modern genome-wide sequencing technologies have recently been used to rapidly discover inversion loci, measure their frequencies across populations, and test for associations with adaptation and speciation, leading to the discovery of large numbers of inversions across a wide range of species [1, 16–18].

Despite the evidence that inversions are common and important for many evolutionary processes, there are still very few examples where both the functional variants and adaptive phenotypes that are controlled by any particular inversion are known [19]. Inversions affect fitness through genetic linkage with variants underlying adaptive traits [20]. These variants could be the breakpoints themselves if they disrupt genes, their promoter regions, or the local chromatin regulation [21, 22], or could be variants located between the breakpoints that are linked due to the recombination suppression. It is generally expected that at least two adaptive variants must be linked to an inversion for it to have a fitness advantage [4]. However, large inversions could contain many functional variants, each potentially affecting multiple downstream phenotypes, and thus can form supergenes [23]. Unfortunately, the recombination suppression that gives inversions their evolutionary advantage also makes traditional QTL mapping and Genome-Wide Association Studies ineffective at resolving the independent effects of the different variants within an inversion locus.

In recent years, gene expression analysis has emerged as a powerful tool to dissect the functional impacts of many types of mutations, including chromosomal inversions [22, 24–26]. RNA sequencing is a very data-rich phenotyping technology that can simultaneously measure tens of thousands of different gene expression values from each experimental sample. The expression of each gene responds to a different combination of transcription factors, gene networks, cellular states, and environmental cues, so measuring gene expression provides an indirect measurement of a wide range of cellular, developmental and physiological characteristics of an organism. These cellular or physiological traits may be critical for adaptation, yet are often neglected in evolutionary studies because they are difficult and costly to measure directly. At the same time, gene expression analysis can be used to scan across an inversion locus gene-by-gene to identify specific genes that have different cis-regulatory genetic control among alleles. If the functional variation captured by an inversion locus operates by directly altering the expression of genes in the inversion, we can identify these genes by their expression changes without relying on recombination. Together, these two types of gene expression analysis may greatly advance our understanding of inversion loci, particularly those that are not feasible to study by other means.

In this study, we applied population genetic and gene expression analyses to study an inversion locus in maize. Maize is an important crop species worldwide and also a powerful model system for studying the mechanisms of recent and rapid local adaptation. Maize (*Zea mays* ssp. *mays*) was domesticated in the lowland Balsas river valley of southwestern Mexico from the lowland-restricted teosinte subspecies, *Zea mays* ssp. *parviglumus* (hereafter *parviglumus*) approximately 9000 years ago [27, 28]. Since domestication, populations of maize have been moved into high altitude environments, and landraces collected today show considerable local adaptation to their home elevation in a range of traits [29]. Interestingly, population genetic scans for loci associated with adaptation to elevation gradients have identified several loci common in highland landraces that have been introgressed from a different subspecies of teosinte, *Zea mays* ssp. *mexicana* (hereafter *mexicana*), which occurs in highland environments [30]. One of these introgressed regions, located at approximately 171.7 to 185.9 Mb of chromosome 4, is a chromosomal inversion known as *Inv4m* [31, 32]. *Inv4m* is mainly found in maize landraces from high elevation regions of Mexico. A recent study observed that *Inv4m* was associated with a three day acceleration of flowering time, the largest effect flowering QTL known [33]. In two different studies of teosinte, *Inv4m* was found to be enriched for SNPs associated with highland adaptive traits [34], and the inversion overlapped with adaptive quantitative trait loci (QTL) controlling leaf pigmentation and macrohairs [35]. However, the precise mechanisms underlying *Inv4m*’s role in maize highland adaptation is not yet known.

The central hypothesis of our study is that *Inv4m* controls multiple traits of adaptive importance in the highlands of Mexico, and that the sensitivity of genome-wide gene expression profiling can help identify these traits and pinpoint the genes within the inversion that are responsible. We expected that large-effect loci within *Inv4m* should be observable as direct cis-acting expression changes on specific genes, and that the expression of these *Inv4m*-located genes will be correlated with expression effects in groups of functionally-related downstream genes. If so, the identities of these downstream genes and the tissue contexts of the *Inv4m*-effects should help us describe the range of physiological and/or cellular effects of *Inv4m*, while narrowing down the list of candidate genes within the locus. If so, this approach would provide a tool for dissecting the function of other inversions in other systems.

We first comprehensively characterized the population-genetics context of the *Inv4m* locus in maize and studied its association with key agronomic traits using dense whole-genome genotyping data of thousands of Mexican landraces. We found that *Inv4m* is highly diverged across the locus, is more closely associated with altitude in the center of maize diversity in Mexico than nearly any other locus in the maize genome, and shows clear patterns of fitness trade-offs indicative of a key role in local adaptation.

We then used experimental crosses to introgress the highland haplotype of *Inv4m* into a common reference maize background so that we could measure the genome wide expression effects without confounds of multiple genetic backgrounds. We repeated the introgression twice from two donor sources to identify effects common to highland haplotypes instead of specific to a particular genotype. We measured expression effects in each of nine different tissues in both high and low temperature conditions to identify as many effects of the locus as possible. Lastly, we scanned inside the *Inv4m* locus for genes with high gene expression correlation with the set of *Inv4m*-regulated genes genome-wide and identified several outlier genes inside the *Inv4m* locus which are candidates for further study.

Our results suggest *Inv4m* regulates a diverse range of biological processes in highland maize, and suggest new hypotheses for candidate physiological and cellullar traits that may be involved in maize highland adaptation. Of particular interest is a signal of up-regulated photosynthetic pathways under cold temperatures and down-regulated photosynthetic pathways under warm temperatures in samples from the youngest developmental leaf that we sampled.

## Results

### *Inv4m* haplotypes are strongly diverged and associated with local adaptation in agronomic traits

Romero-Navarro *et al* [33] identified *Inv4m* as a large-effect QTL in a multi-environment trial of landrace hybrids grown in Mexico, and observed that *Inv4m* frequencies were highly differentiated between highland, subtropical and lowland tropical populations. We repeated this analysis to study the geographic distribution of *Inv4m* in more detail.

**Fig 1:**
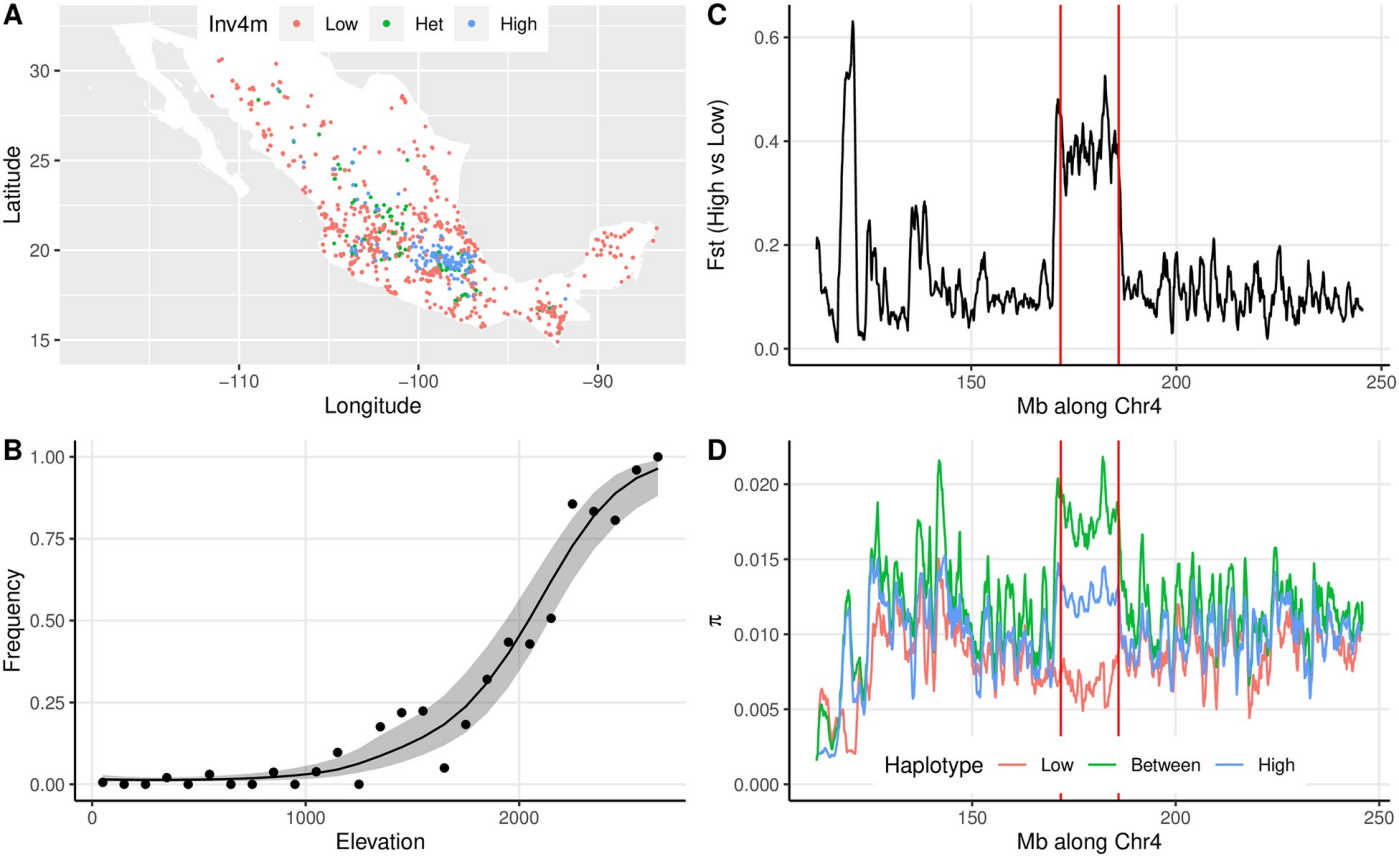
Association of *Inv4m* genotype with environmental factors and agronomic traits. **A**. Geographic locations for each of the 1757 Mexican plants genotyped by GBS, colored by their imputed genotypes at *Inv4m*. **B**. Association of *Inv4m* and elevation. Each point shows the mean frequency of the “High” allele at the *Inv4m* locus among plants from landraces collected in each 100m bin. The ribbon shows a loess fit (± 2SE) to the logit-transformed frequencies weighted by the number of landraces in each elevation bin. Bins with fewer than 10 landraces were excluded (those with elevation >2700m). **C** *F*_*ST*_ between 4 landraces from Mexican highlands and 5 landraces from Mexican lowlands [36] across an ≈ 130*Mb* region of chromosome 4. Points show average *F*_*ST*_ across 1Mb regions with a step size of 100Kb. **D**. Average pairwise nucleotide diversity estimates across the same regions within each population and between the two populations. The boundaries of *Inv4m* from [31] are denoted by vertical lines.

We determined the *Inv4m* genotype of 4845 maize plants from the SeeD-maize GWAS panel using published unimputed genotype-by-sequencing (GBS) data. Of these lines, 707 were homozygous for the inverted minor haplotype, and 351 were heterozygous. Of the 585 plants carrying at least one allele of the minor haplotype and with complete with geographic information, all but 7 were from Mexico, with the majority collected from the central highlands (Fig 1A). Therefore, to assess evidence of local adaptation, we modeled the association of *Inv4m* genotype with elevation among the 1757 Mexican plants. In Mexico, 1186 and 381 plants were homozygous for the alternate non-inverted and inverted haplotypes, respectively, and 190 were heterozygous, a distribution that significantly differs from Hardy-Weinberg expectations (D = -252.0, p = 1.91e-197). Genotypes at *Inv4m* were strongly associated with elevation, as previously reported [33] (Fig 1B).

While useful for genotyping individuals for the major haplotypes at *Inv4m*, this GBS dataset was not appropriate for quantifying genetic divergence within or among haplotypes because data are only available for variable sites, and these sites may be impacted by ascertainment bias against the rarer *Inv4m* haplotype. We therefore used previously published whole-genome-sequencing data from 4 highland and 5 lowland maize landraces previously shown to carry alternate alleles at *Inv4m* to calculate nucleotide diversity across the *Inv4m* locus [36]. These data showed that the *Inv4m* locus is considerably more diverged between the highland and lowland populations than flanking regions along chromosome 4 (Fig 1C), with average *F*_*ST*_ approximately 0.38 across the locus. Based on within and between-population pairwise nucleotide diversity statistics (*π*) the highland allele is more diverse than the lowland allele inside *Inv4m*, and more diverse than much of the surrounding genomic regions along chromosome 4 (Fig 1D). Therefore, the two haplotypes are relatively old and have shared very little gene flow for thousands of generations.

The near-fixation of alternative alleles of *Inv4m* along the elevation gradient in Mexico strongly supports the idea that *Inv4m* is under selection and contributes to local adaptation. But this doesn’t itself help identify *why*, i.e. what traits are controlled by *Inv4m* and are responsible for this selection? Romero-Navarro *et al* [33] identified strong associations of SNPs inside *Inv4m* with flowering time in at least some of their field trials. However, none of the individual SNP markers reported in this study were perfectly associated with the *Inv4m* genotype, and the association with one trait does not preclude it having additional affects on other adaptation-related phenotypes. Using data from the same SNP markers and field trials, Gates *et al* [37] identified associations between SNPs inside *Inv4m* and the *plasticity* to elevation for five of six agronomic traits: Days-to-Anthesis, the Anthesis-Silking Interval (ASI), Grain Weight-per-hectare, Bare cob weight, and Field Weight (but not Plant Height).

**Fig 2:**
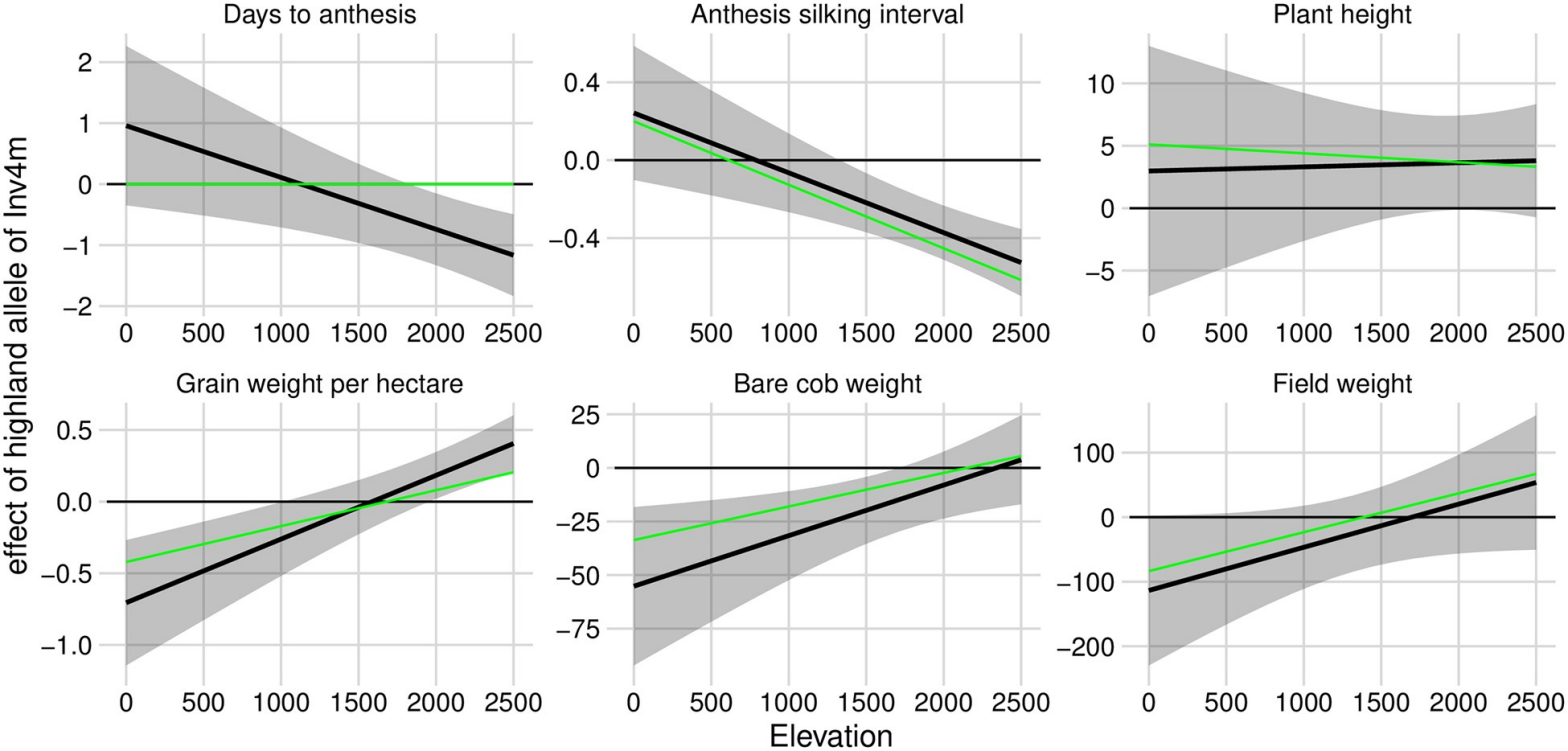
Association of *Inv4m* genotype with agronomic traits in field trials changes as a function of elevation. We modeled each trait as a function of *Inv4m* genotype, trial elevation, and tester line, with controls for main effects and responses to elevation of the genomic background. Black lines and ribbons show estimates of the effect of the highland allele of *Inv4m* as a function of trial elevation ± 2SE, based on conditional F-tests at the REML solutions of the random effect variance components. Green lines show estimates of the *Inv4m* effect in a model that additionally included effects of Days-to-Anthesis on the focal trait within each trial.

We refined these observations by re-running the genetic association models reported by Gates *et al* [37] to extract estimates of the effect of the inverted allele of *Inv4m* as a function of the elevation of the field trial. For each trait, except for Plant Height, the inverted (highland) haplotype of *Inv4m* was associated with trait variation in the direction consistent with local adaptation: earlier flowering, reduced ASI, and increased yield components in highland trials, while the opposite effect was observed in lowland trials (Fig 2). The highland haplotype was weakly associated with greater plant height across elevations, but the relationship was not significant (p = 0.11 for the main effect). These results are consistent with this single locus causing antagonistic pleiotropy [38] across elevation environments, which would explain the strong divergence in allele frequencies across elevation. The relationship between *Inv4m* genotype and these traits was not simply an indirect effect on flowering time; each relationship remained qualitatively the same even after accounting for the the effect of Days-to-Anthesis separately within each trial. The inversion appears to independently control both flowering and yield traits which is consistent with the hypothesis that *Inv4m* contains multiple important variants that each contribute to phenotypic differences between lowland and highland maize populations [33, 37].

However, while compelling, due to the strong population structure in this diversity panel, we caution that the associations of *Inv4m* with the agronomic traits is still preliminary. *Inv4m* genotypes are highly correlated to overall genetic ancestry (as measured by PC1 calculated using all other chromosomes except chromosome 4) within Mexico. We corrected for ancestry using genome-wide kinship (again excluding chromosome 4), but if this correction was incomplete, it may have lead to false-positive associations with *Inv4m*. An alternative explanation for the phenotypic associations above is that each trait has a polygenic basis, with small-effect loci distributed throughout the genome, each of which has subtle allele-frequency differences across elevations. Therefore, any associations of traits with *Inv4m* in this population could be driven by the combination of many loci genome-wide all correlated with this dominant axis of genomic divergence. To confirm that *Inv4m* in fact causes important phenotypic effects we must experimentally break the association between *Inv4m* and the rest of the genome through experimental crosses, as we describe next.

### Introgression of highland alleles of *Inv4m* regulate diverse phenotypes

**Fig 3:**
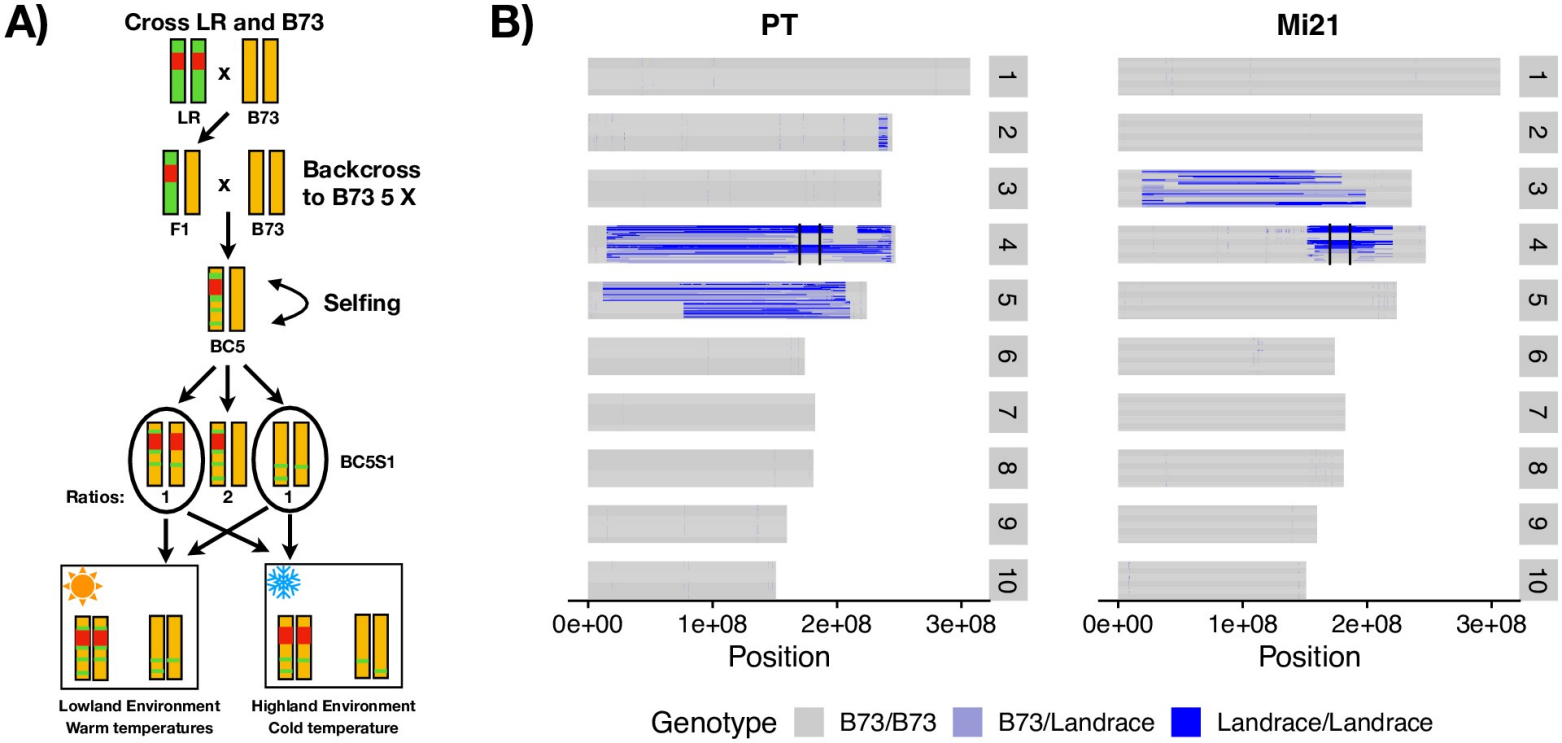
Plant families segregating for *Inv4m*. A) Graphical overview of crossing scheme and experimental design. Red represents the high haplotype of *Inv4m*, green represents the landrace genome, and gold represents the B73 genome. We used the same crossing scheme design for both donor landraces. B) Genotypes called from RNA sequencing data across the maize genome. In each panel, horizontal rows of each chromosome represents the genotypes of a single plant from either the PT-derived or Mi21-derived families, with blue representing landrace alleles and grey the B73 background. Within each family, plants are sorted first by maternal plant (2 per family) and then by genotype at *Inv4m*. Vertical black lines on chromosome 4 delineate the location of *Inv4m*.

We generated two sets of segregating families for alternative alleles of the *Inv4m* locus by crossing the reference line B73, which carries a lowland haplotype of *Inv4m*, to two highland landrace donors (called PT and Mi21). The two families were used as replicates of the effect of the highland haplotype of *Inv4m* to ensure that *Inv4m*-associated effects that we identified were not specific to a single highland landrace. Progeny were backcrossed to B73 for 5 generations and then selfed to generate a population of plants that were largely homozygous B73 across the genome, but segregated for the lowland and highland haplotypes at the *Inv4m* locus (Fig 3). We grew a total of 360 plants in growth chambers simulating two temperature environments (warm: 32C/22C and cold: 22C/11C), genotyped each plant using a diagnostic CAPS marker for *Inv4m*, and selected 81 plants homozygous for either the High or Low haplotypes for further analysis.

#### Phenotype measurements

We sampled multiple tissues (Table 1) from the 81 selected plants at either the V1 or V3 developmental stage to measure the effect of *Inv4m* on genome-wide gene expression (S1 Fig). We selected nine total tissues (S2 Fig) with the goal of capturing as much variation in expression profiles as possible in maize seedlings, given the experimental constraints on how large we could let the plants grow in our growth chambers, based on the maize gene expression atlas [39]. Of the 432 tissue samples collected (nine tissues × two temperature treatments × two segregating families × two *Inv4m* arrangements × three biological replicates × two experimental replicates), we excluded 53 samples with fewer than 100,000 reads, leaving a total of 379 samples. We detected a total of 23,428 genes with 10-or-more counts in at least a third of the samples (with an average of 17,016 genes per tissue) for a total of more than 306,000 gene expression traits. We used multi-dimensional scaling (MDS) analysis to visualize the overall transcriptome variation among samples (S3 Fig). Samples clustered predominantly by tissue, with an additional slight separation by temperature treatment, but no visible separation by family or genotype at the *Inv4m* locus. This was expected because only ∼ 7% of the genomes differed among samples. Among tissues, all leaf samples except the S2 leaf base formed one major cluster, the two root tissues formed a second cluster, and the stem and SAM and S2 leaf base tissues formed separate individual clusters in the MDS plot.

**Table 1:** Developmental stage and description of tissues sampled for gene expression analysis.

Developmental Stage	Tissue	Description
V1	Root	Primary root
V3	Root	Primary root
V3	SAM	Stem Apical Meristem
V1	Leaf	Pooled leaf tissue
V3	Sheath	Leaf sheath
V3	Leaf base	5 cm of leaf base
V3	Leaf tip	5 cm of leaf tip
V3	S2 leaf base	5 cm of leaf base
V3	S2 leaf tip	5 cm of leaf tip

In addition to gene expression, we quantified the effect of *Inv4m* on seedling emergence in each temperature treatment separately. Emergence times in the cold chamber averaged approximately 9 days, and plants with the High haplotype of *Inv4m* emerged 0.75 and 0.35 days earlier on average in the PT and Mi21 families, respectively (S1 Table). In the warm chamber, the average emergence time was approximately 4 days, and the High *Inv4m* haplotype had no detectable effect (S1 Table).

#### Quantification of residual landrace alleles in BC_5_S_1_ plants

As a preliminary step in identifying *Inv4m*-associated traits in this population, we characterized each plant for the extent of residual landrace genome that remained after the five generations of backcrossing to B73. This residual landrace genome could bias both our expression results due to mapping biases, and confound our analysis of the effect of *Inv4m* if these residual regions contain functional variants between each landrace and B73.

**Fig 4:**
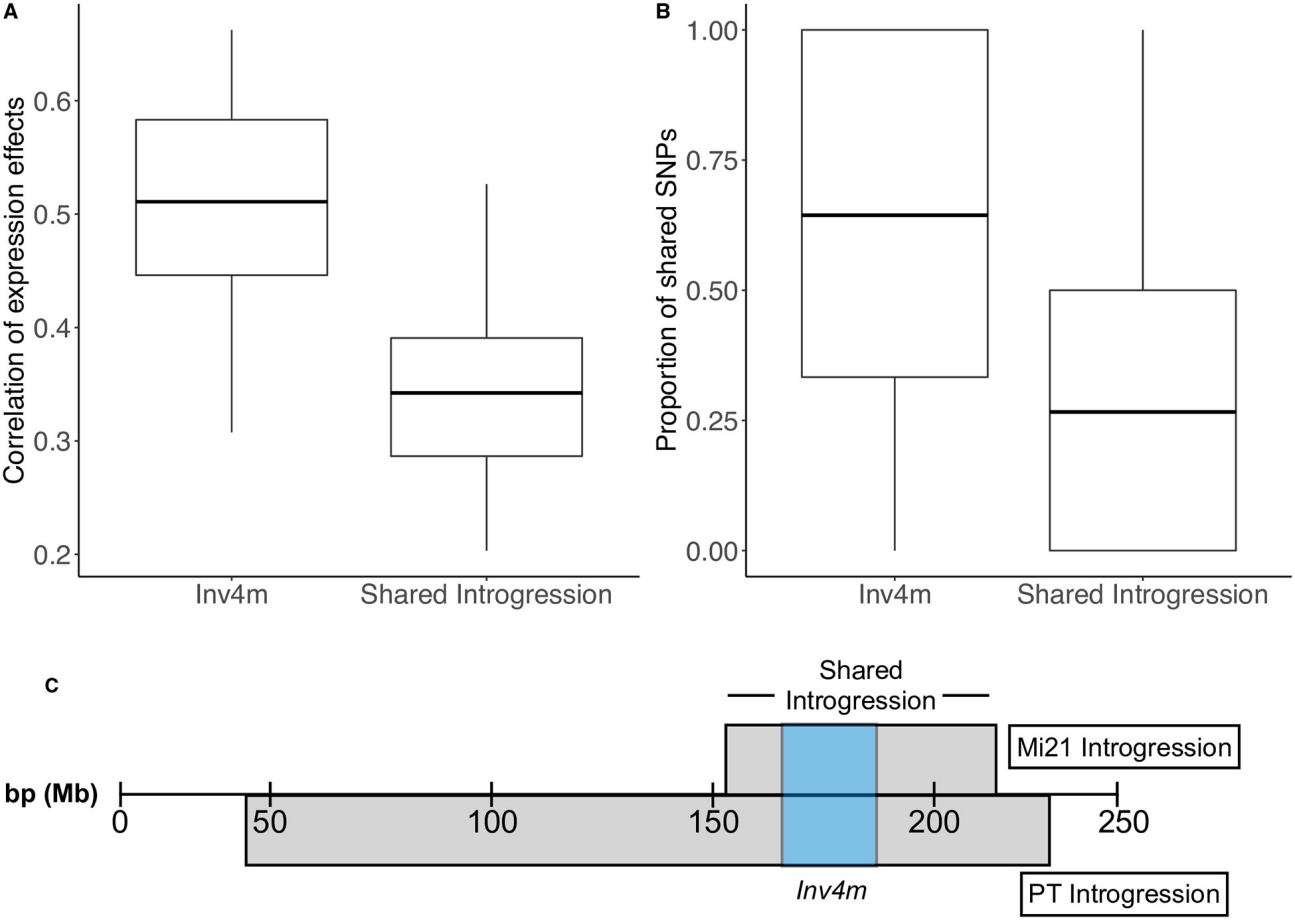
Sequence and expression divergence between landrace donors is greater outside of the *Inv4m* boundaries than within. A) Boxplots show the correlation between genetic and expression divergence between the two donor populations for genes inside *Inv4m* or outside *Inv4m* but in the region of shared introgression. Correlations were measured separately in each of the 18 tissue:temperature combinations. B) Boxplots show the proportion of shared SNPs per gene for genes inside or outside *Inv4m*. C) Diagram of PT and Mi21 introgressions containing *Inv4m* on chromosome 4.

We used RNAseq reads to identify residual landrace alleles at every expressed gene in each of the BC_5_S_1_ plants. We observed residual alleles in large regions flanking the *Inv4m* locus in plants from all families. In the PT segregating family, residual PT alleles were observed in a 57Mb window surrounding *Inv4m*. In the Mi21 family, residual Mi21 alleles were observed in an 18Mb region surrounding *Inv4m* (Fig 4A). The PT family also segregates for a large paracentric region of residual donor alleles on chromosome 5 and a small region on chromosome 2, and the Mi21 family segregates for a large paracentric residual region on chromosome 3 (Fig 3B). Beyond these large contiguous blocks, we identified another 821 and 52 genes in the PT and Mi21 families, respectively, that harbored high-confidence SNPs in the RNAseq data, yet were not contiguous with any of the large residual introgression regions (Fig 3). It is unlikely that there was sufficient recombination in the BC_5_ populations to generate these independent blocks; rather these genes likely have moved genomic coordinates in the landraces relative to B73, and actually reside inside one of the large introgression regions [40, 41]. None of the residual genes had genotypes that were perfectly correlated with genotypes at the *Inv4m* haplotype, so we excluded them all from further analysis (S4 Fig).

We also used the RNAseq reads that did not map to the B73 genome to search for genes that may be present in the High haplotype of *Inv4m* but not in the Low haplotype, and thus may have been missed by our analysis. We assembled all un-mapped reads from samples carrying the PT or Mi21 haplotype at *Inv4m* using Trinity, and searched for de-novo transcripts with evidence for expression only in the samples carrying the High haplotype. We found 772 candidate transcripts. However, all were very lowly expressed. Only five had estimated transcript counts of at least 10 summed across all *PT* or *Mi21* samples, and none had estimated transcript counts of at least 10 in two or more samples per family. Therefore, we found no evidence that the High haplotype of *Inv4m* carries high-expressed genes that are not present in the Low haplotype.

In the segregating families there were a total of 7,236 genes with PT alleles, and 4,095 genes with Mi21 alleles, of which 355 were within *Inv4m*. Of all genes from the landrace donor which were highly correlated with the *Inv4m* genotype (*ρ* > 0.5), 14% and 29% resided inside *Inv4m* in the PT and Mi21 families respectively. Therefore, many associations of traits with the *Inv4m* haplotype that we observe in one segregating family may be caused by functional variants inherited from the PT or Mi21 landraces in regions flaking the inversion, rather than functional variants inside the inversion itself. Variants specific to either the PT or Mi21 allele of *Inv4m* cannot be responsible for the adaptive value of the inversion. However, since population level variation in the regions flanking *Inv4m* was not correlated with *Inv4m* genotype (Fig 1A), *Inv4m*-associated effects that replicate in our two segregating families were likely caused by alleles located within *Inv4m* and which were common to at least these two samples of the highland haplotype.

We tested the likelihood that gene-regulatory variation was due to *Inv4m*, or the shared residual introgression flanking *Inv4m*. We quantified the genetic and expression divergence between families using plants that were homozygous either for the PT or Mi21 *Inv4m* alleles. The proportion of shared SNPs between PT and Mi21 families within *Inv4m* was significantly higher than in the flanking introgressed regions (Fig 4B, t = 10.937, df = 319.93, p-value < 2.2e-16). The same pattern occurred for gene expression variation attributed to landrace genotypes for each gene: the landrace genotype effect on genes inside *Inv4m* were more correlated than landrace genotype effects in flanking regions (Fig 4A, t = 5.1793, df = 33.162, p-value = 1.073e-05) across the 18 tissue:temperature combinations. Therefore, gene regulatory effects associated with *Inv4m* that replicate across the two donor populations are more likely to be caused by functional variation inside *Inv4m* rather than in residual landrace DNA present in each population (Fig 4). We also quantified the correlation between genetic dissimilarity and expression divergence between *Inv4m* sources (Mi21 and PT) across the shared introgressed region containing the inversion. We found that genetic divergence between Mi21 and PT was higher outside of the inversion that within the breakpoints, and that expression divergence was higher outside of the inversion boundary. We calculated expression divergence as the average log-fold change in expression averaged across tissue and temperature combinations between Mi21 and PT (S5 Fig). These two separate measures of divergence were significantly positively correlated (r = 0.80, p = 2.2e-16, df = 76).

#### Effects of *Inv4m* on genome-wide gene expression

We measured the effect of the highland allele on each gene within each segregating family for all 18 tissue:temperature conditions to quantify *Inv4m*-associated gene expression effects, and used *multivariate adaptive shrinkage* [42] to increase precision and power by sharing information across the 18 conditions. Then, we classified *Inv4m*-effects as being successfully replicated if the sign of the effect was the same in both segregating families, and was significant (as defined as a local false sign rate (*lfsr* less that 5%) in both segregating families.

We excluded genes in genomic regions with residual landrace alleles to avoid biased effect estimates. 11,842 and 12,482 genes were associated with *Inv4m* in the PT and Mi21 segregating families, respectively, in at least one tissue:temperature condition using a 5% local false sign rate (*lfsr*) threshold for significance (Table 1), with 1,645-5,532 and 145-6,208 genes per condition in the PT and Mi21 families respectively. Of these genes, 39-607 per tissue replicated across both donor families, totaling 8–41% of the differentially expressed genes in the PT family, and 11–38% of the differentially expressed genes in the Mi21 family.

In contrast, we found little evidence for strong genotype-environment interaction effects for *Inv4m* on gene expression. We detected 46-435 genes per tissue in the PT family and 127-1408 genes per tissue in the Mi21 family with significant genotype-treatment interactions at a 5% *lfsr* threshold. However only 1-38 genes per tissue replicated between the two families (Table 2), suggesting that the majority may be allele-specific and therefore we were not confident that they were representative of the high haplotype of *Inv4m* in general.

**Table 2:** Number of differentially expressed genes in each population and their overlap using a local false sign rate threshold of 5% across tissues and temperature treatments.

	Cold			Warm			G × E		
Tissue	PT	Mi21	Shared	PT	Mi21	Shared	PT	Mi21	Shared
V1 Leaf tissue	2504	1474	151	1741	1332	92	46	127	1
V1 Primary root	2124	3732	193	3014	2302	307	57	285	2
V3 leaf base	1671	3990	262	1645	3065	105	47	516	5
V3 leaf sheath	2535	4400	282	2382	3815	389	268	641	17
V3 Leaf tissue	2170	145	39	3176	1652	152	435	279	26
V3 Primary root	5532	3291	607	1653	3004	84	48	367	1
V3 S2 leaf base	2833	6208	306	2916	5135	531	378	1408	38
V3 S2 leaf tip	2846	2342	273	3728	3003	202	268	683	20
V3 Stem and SAM	2497	4370	264	2584	2954	77	208	1021	23

### *Inv4m-regulated* genes are enriched for diverse biological processes

The list of consensus *Inv4m-regulated* genes are reporters of the effect of *Inv4m*, and were distributed across the genome with no visible clustering by chromosome. To summarize these genome-wide responses to *Inv4m* genotype and explore whether *Inv4m* regulates one, or many, separate processes, we tested for enrichments of Gene Ontology (GO) terms within the sets of *Inv4m-regulated* reporter genes. For each tissue:treatment experiment, we separately quantified GO term enrichments among sets of reporter genes that were up-regulated, down-regulated, or regulated in either direction by *Inv4m*. We identified a total of 564 enriched categories overall, with 0-114 signifiantly enriched categories per tissue:temperature treatment at a 5% FDR. These GO terms provide candidate descriptors of the global effects of *Inv4m*. However, GO terms are highly redundant, so to reduce the number of categories and combine results across all tissue:temperature treatments, we collapsed terms into clusters by semantic similarity [43] and selected the most-enriched term across all tissue:temperature treatments in each cluster to label the sets of effects. After this filtering step, we ended with a list of twenty-one representative GO terms describing *Inv4m* effects across the three main GO ontologies: two cellular component terms, one molecular function term, and eighteen biological processes terms (Table 3).

**Table 3:** Representative Gene ontology (GO) terms for *Inv4m*-regulated genes. A universal enrichment analysis was conducted on each tissue and temperature and directional (up-regulated, down-regulated, or both) combination for *Inv4m*-regulated genes. Terms were then ranked by enrichment score and grouped by a semantic similarity score of higher than 0.5. The top term in each semantic similarity group selected to represent the group.

ID	Ontology	Term	minq	maxRatio
GO:0015979	BP	photosynthesis	2.27e-14	3.18e-01
GO:0034470	BP	ncRNA processing	4.55e-03	1.35e-01
GO:0006006	BP	glucose metabolic process	5.30e-03	1.20e-01
GO:0006733	BP	oxidoreduction coenzyme metabolic process	2.97e-05	1.15e-01
GO:0019682	BP	glyceraldehyde-3-phosphate metabolic process	2.85e-07	1.10e-01
GO:0009657	BP	plastid organization	1.75e-05	1.10e-01
GO:0072524	BP	pyridine-containing compound metabolic process	3.50e-05	1.05e-01
GO:0008654	BP	phospholipid biosynthetic process	3.90e-05	1.05e-01
GO:0044272	BP	sulfur compound biosynthetic process	1.59e-04	1.05e-01
GO:0007017	BP	microtubule-based process	3.64e-03	9.57e-02
GO:0006090	BP	pyruvate metabolic process	1.08e-03	9.00e-02
GO:1903047	BP	mitotic cell cycle process	5.81e-03	8.13e-02
GO:0090698	BP	post-embryonic plant morphogenesis	6.84e-03	8.13e-02
GO:0042440	BP	pigment metabolic process	9.73e-03	7.50e-02
GO:0033013	BP	tetrapyrrole metabolic process	3.96e-04	7.00e-02
GO:0009630	BP	gravitropism	8.26e-04	6.06e-02
GO:0035304	BP	regulation of protein dephosphorylation	1.11e-04	5.50e-02
GO:0022900	BP	electron transport chain	3.23e-03	5.50e-02
GO:0004004	MF	ATP-dependent RNA helicase activity	8.66e-03	8.16e-02
GO:0009534	CC	chloroplast thylakoid	2.27e-14	4.55e-01
GO:0044391	CC	ribosomal subunit	4.66e-03	9.15e-02

Among the 564 enriched GO terms, a set of 8 terms involving photosynthesis stood out as being the most strongly enriched among the candidate *Inv4m*-regulated genes. These categories are largely over-lapping in their gene content, so we selected the term: *GO:0009534: Chloroplast thylakoid* from the Cellular Component ontology as a representative label for this *Inv4m* effect. Genes annotated with this term tended to be up-regulated by *Inv4m* in the basal (developmentally immature) section of the S2 leaf in the cool temperature treatment, but down-regulated in this tissue in the warm temperature treatment (S6 Fig). *Inv4m*-associated effects on these genes were present in one or the other of the families in other tissues, but with inconsistent effect directions so we cannot conclude that these were driven by *Inv4m* itself rather than by other linked regions of residual genomic introgression present in each family (S6 Fig). These results suggest that *Inv4m* may regulate some aspect of chloroplast development or activity. To test for a change in chloroplast activity, we inspected the expression of chloroplast-expressed genes. Genes expressed in the chloroplast are inconsistently represented by our RNAseq libraries because their transcripts are not generally polyadenylated [44]. However we were able to quantify expression of 39 chloroplast genes, 8 of which were differentially expressed in at least one tissue. Overall, plants with the high *Inv4m* haplotype had higher expression of chlorophyllic genes in the plastid genome in the tip of the S2 leaf the cool temperature, and lower average expression in the warm temperature. This mirrors the patterns we observed for chloroplast-related nuclear genes, but only in the more developmentally mature region of this leaf (S6 Fig).

Since the phenotypic association of *Inv4m* with flowering time was so strong, we collected a list of genes with known roles in regulating flowering from two sources [45, 46] and used this list as another term to test for *Inv4m*-effects. We removed genes that were within any landrace introgression block, and listed any gene regulated by *Inv4m* that occurs in either of the flowering gene lists (S3 Table). While several genes from this list were regulated by *Inv4m*, as a whole, this category was not enriched for *Inv4m* effects relative to the rest of the genome (p = 0.4).

### Identifying candidate causal alleles within *Inv4m*

As a final step, we attempted to use the RNAseq data to identify candidate genes for the effect of *Inv4m* itself. The causal variants responsible for *Inv4m*’s effect must lie within or at the boundaries of *Inv4m*. And if the causal variants operate by altering the expression of *Inv4m* genes, we may be able to detect these by inspecting for *Inv4m*-effects on *Inv4m*-located genes. Furthermore, if the expression of these genes were responsible for the effects of the *Inv4m* locus, we would expect that the expression of these genes would be a significant predictor of at least a subset of the *Inv4m*-regulated reported genes (genome-wide), even after accounting for the genotype at the *Inv4m* locus.

**Fig 5:**
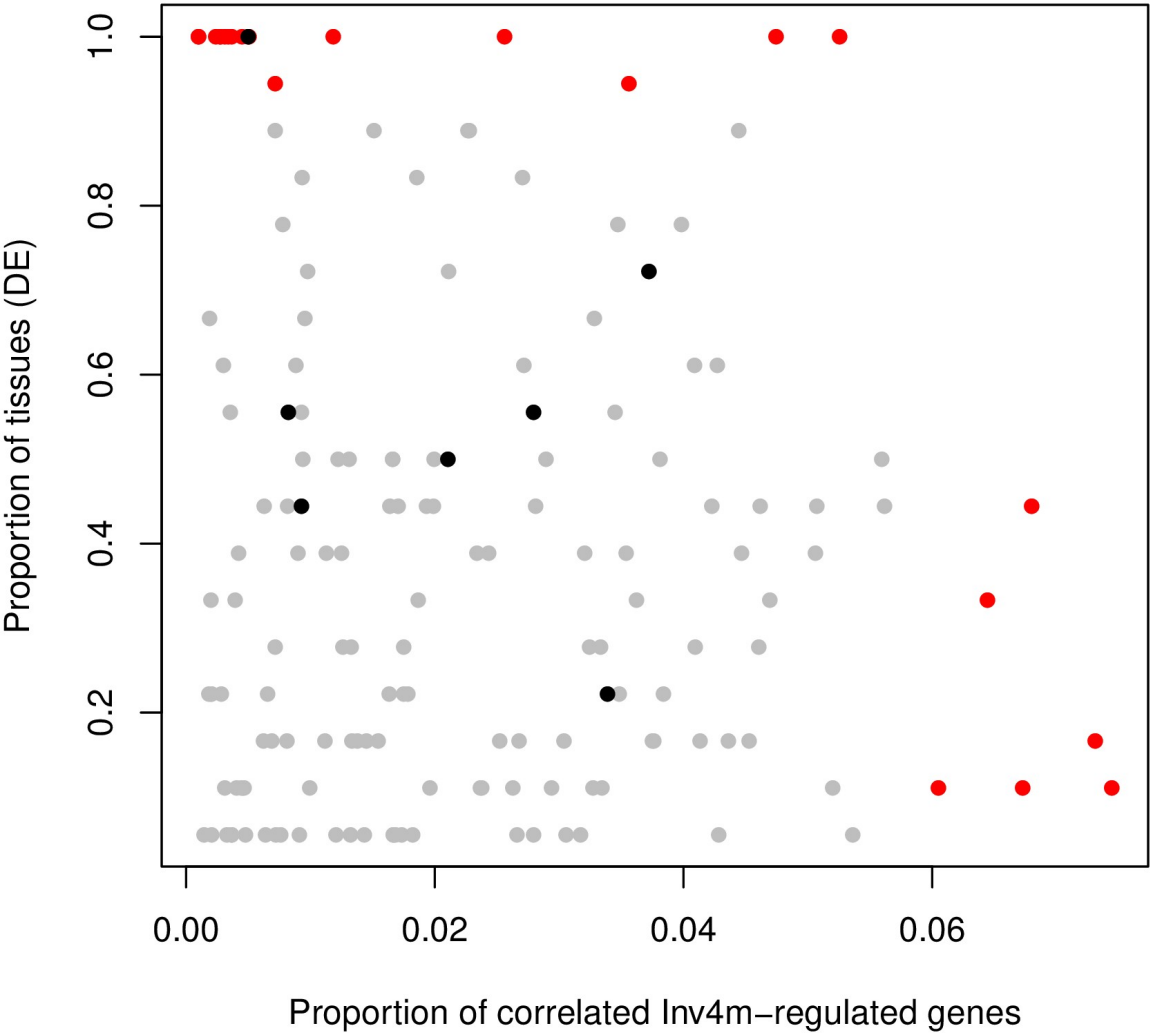
Scatter plot of candidate gene scans within *Inv4m*. Each point represents one of the 160 genes inside *Inv4m* that were both differentially expressed and correlated with at least one *Inv4m-regulated* gene. The x-axis represents the proportion of *Inv4m-regulated* genes correlated with the *Inv4m*-gene (p-value< 0.05). The y-axis represents the proportion of tissues in which the *Inv4m*-gene is differentially expressed (lfsr< 0.05, and regulated in the same direction in both populations.) Red points are correlated with more than 6% of *Inv4m-regulated* genes on average, or are differentially expressed in more than 90% of the tissues in which it was expressed. Black points are transcription factors.

To identify candidate genes within *Inv4m*, we measured the *Inv4m* genotype effect (i.e. *cis* effect) on each of the annotated genes located inside the *Inv4m* locus, and the correlation between the expression of these *Inv4m*-genes and all *Inv4m-regulated* genes outside of the locus (a total of 4642 unique genes, with a range of 89-713 genes per tissue; Fig 5). We repeated this analysis in each of the 18 tissue:temperature treatments. For *cis*-genotype effects to be counted, we required the effects to be significant (*lfsr* < 5%) and in the same direction between the two donor populations.

Overall, of 355 annotated genes within the boundaries of *Inv4m*, 224 were expressed in both donors. Of those, 182 were differentially expressed in the same direction in both donors in at least one condition, and 160 of those were significantly correlated with at least one *Inv4m-regulated* gene located on other chromosomes used to measure the global *Inv4m effect*, even after accounting for the effect of *Inv4m* itself (Fig 5). Of these, 6 genes in particular stood out as being correlated with a large number (> 6%) of the reporter genes: Zm00001d051885, Zm00001d051929, Zm00001d051995, Zm00001d052075, Zm00001d052101, Zm00001d052224, and 17 were differentially expressed between *Inv4m* alleles in 90% or more of the tissue:temperature combinations. Finally, 7 of the 160 candidate *Inv4m* genes were transcription factors. One of the seven transcription factors, Zm00001d052054, was differentially expressed in the same direction in every tissue, and is predicted to be a sequence-specific DNA binding transcription factor (S2 Table). We did not observe an enrichment of *Inv4m*-related genes near the inversion breakpoints, which would have implicated the breakpoints themselves as functional variants for *Inv4m*.

The screening steps above excluded one *a priori* candidate gene within *Inv4m*: the microRNA MIR172c, which is located inside *Inv4m* at coordinates: 4:174154928-174155050. MIR172 has known roles in regulating developmental transitions as well the development of pigmentation and macrohairs in maize [47] and therefore was a strong candidate given that increased pigmentation and macrohair density is common in highland landraces and *Inv4m* overlaps a QTL for pigmentation and macrohairs in teosinte [35]. The expression of the pre-miRNA 172c was too low for us to measure differential expression in all but the leaf sheath tissue, and was not associated with *Inv4m* genotype in that tissue. However, of 31 genes that are predicted targets of miR172, 6 were *Inv4m-regulated* in both populations in at least one tissue. Of these, four were up-regulated by the High haplotype of *Inv4m*, one was down-regulated, and one was upregulated in one tissue and downregulated in another tissue (S4 Table).

## Discussion

Chromosomal inversions are a common and evolutionarily important form of structural variation in the genomes of many taxa, and have frequently been associated with adaptive traits [1, 48, 49]. However, many questions remain about how inversions form, spread within and among populations, and influence the evolutionary history of genomes over both short and long time-scales [4, 19, 50]. One key question is whether inversions generally contain only two important functional variants, or if they capture many, perhaps because they act as a sieve for new mutations [51–54], and whether these variants contribute to the same trait, or independently regulate multiple traits that may all be adaptive. Our study focused on an inversion polymorphism that is associated with local adaptation of maize landraces to highland environments in central Mexico, and which has a complicated evolutionary history involving introgression into highland populations from maize’s wild relative, *Z. mays* ssp. *mexicana*. We studied both the population genetics and functional effects of the alternative haplotypes at this locus, and found evidence that the two major haplotypes are effectively isolated from each other, presumably by both recombination suppression and selection, and that the allelic differences between the haplotypes appears to control multiple selectively important traits including flowering time, yield components, photosynthetic development, and a suite of other molecular traits. However, we remain unclear about where within *Inv4m* the key functional variants lie, how many there are, and when these variants arose (i.e. before or after the inversion itself was created, *e.g.,* [52, 55, 56]), and so further work on this locus in maize is needed.

Despite their importance, functional studies of inversion polymorphisms remains challenging. For *Inv4m* in particular, we were faced with at least three key challenges: 1) that recombination among the haplotypes is very rare or non-existent, so forward genetics approaches like fine-mapping are not feasible; 2) since *Inv4m* is strongly diverged among populations, it is also highly correlated with genetic ancestry genome-wide and so false-positive phenotype associations are likely in diversity panels, and 3) as a large locus containing >300 genes, many traits are likely controlled by functional variants between the haplotypes, and it is not clear which traits are important for selection and which are not. In this study, we explored how fine-scale population genetic data and large-scale gene expression analysis could help tackle these challenges.

Ultimately, identifying specific functional loci within *Inv4m* will require experimental mutagenesis or other genetic perturbations within the locus. However, we aimed to begin to characterize the diversity of functional variants in this locus using gene expression analysis. We used gene expression in four distinct ways to dissect the functional variation captured by *Inv4m*: 1) by analyzing genome-wide gene expression responses to *Inv4m* to mine for phenotypic effects across >300,000 traits; 2) by analyzing local *cis*-regulatory effects of the locus on the 355 genes within *Inv4m*; 3) by analyzing the co-expression between *Inv4m* genes and the rest of the genome; and 4) by assembling un-mapped transcripts into de-novo gene models. The first analysis provided a detailed phenotypic dissection of the total effects of the *Inv4m*, and showed that there are likely many distinct components to the cellular and physiological effects of *Inv4m*. The second analysis provided an estimate of the density of functional variants within the *Inv4m* locus: we detected likely *cis*-regulatory variation affecting 160 genes. The third analysis showed that many of these cis-regulatory variants may have functional consequences beyond the immediate genes they regulate. The fourth analysis did not give any compelling results, but may be useful in other systems. By studying gene expression effects of *Inv4m* in two relevant environmental contexts (warm and cool temperatures) and across nine distinct tissues, we aimed to maximize our ability to discover developmental and phenotypic effects of the locus. It is certainly possible that we missed important phenotypic effects of *Inv4m* by sampling only tissues on young plants—effects on pathways specific to reproductive tissues were likely missed. However, we selected the nine tissues based on the published maize gene expression atlas [39] so as to capture as much variation as possible in expression profiles, given the experimental constraints on how large we could let the plants grow in our growth chambers.

The strongest Gene Ontology enrichment signals among *Inv4m*-regulated genes were in terms related to photosynthesis and chloroplast development. High elevation adaptation typically requires that plant populations adapt to lower temperatures and shorter growing seasons. For example, in teosinte, stomata density are reduced in highland populations which reduce transpiration in the more arid environments [34]. Compensatory mechanisms are often required to handle the overall reduced enzymatic activity and lower membrane permeability associated with chilling temperatures, especially those associated with photosynthesis [57]. Low temperatures can lead to photoinhibition, where excess electrons from photosystem II bind with Oxygen and produce reactive oxygen species [57]. In our experiment, plants from both *Inv4m* genotypes had increased expression of genes in photosynthesis networks in the cold. However, plants with the high *Inv4m* haplotype had even higher expression in cool temperatures, and lower expression in the warm temperature. The interaction between genotype and temperature may in part explain the adaptive trade-off of this inversion, and the correlation in frequency with elevation, as lower photosynthetic expression in warm conditions may be detrimental in lowland environments. Previous studies have shown that planting maize in cold conditions delay seedling emergence, reduced photosystem II efficiency, chlorophyll content and growth rate [58, 59]. Our findings that *Inv4m* increases PSII and gene expression of chlorophyll genes in the cold may help explain its prevalence in the highlands, and absence in lowlands. Our results also provide molecular evidence of early observations made 40 years ago suggesting that one of the main adaptive advantages of highland maize is the ability to perform active photosynthesis at low temperatures characteristic of highland environments [60].

However, our expression results also show that *Inv4m* affects many disparate biological processes in young maize tissues. We also found evidence of effects on mRNA and protein processing around the nucleus (nuclear transport and import, and the pre-ribosome), epigenetic regulation, cell-cycle processes, metabolism, and development. The majority of the *Inv4m*-regulated genes and summary GO terms were identified in only one or a few tissues, further demonstrating the multifaceted role of this locus. Both flowering and yield are highly complex traits that are affected by many aspects of development, physiology, and stress responses, and so the mechanistic links among these traits may not be obvious [61]. We looked more specifically at *a priori* candidate genes for flowering and yield traits both inside and outside *Inv4m* and found possible effects on several of these genes, but no strong enrichment of *Inv4m* effects on either class.

Among the genes within *Inv4m*, over 70% of those expressed high enough to measure showed evidence of *cis*-regulatory variation, while 45% of landrace genes outside of *Inv4m* were consistently *cis*-regulated. While some of these genes may share regulatory elements, it is likely that the majority of these genes are affected by independent genetic variants. This suggests that the two haplotypes of *Inv4m* harbor a large number of functionally relevant genetic differences. However, does *Inv4m* harbor more functional variants than any other similarly sized introgression among maize landraces? To test this, we compared the number of genes (genome-wide) correlated with *Inv4m* in each segregating family to the number of genes that show similar expression in both donor families. Genes that are regulated similarly in both populations are those we believe are truly affected by *Inv4m*, while the remainder are likely regulated by PT or Mi21 alleles that reside in residual flanking regions outside of *Inv4m*. In both populations, the proportion of *Inv4m* candidates among all *Inv4m*-correlated genes is roughly similar to the relative sizes of *Inv4m* to the whole chromosome 4 introgression in each population. This suggests that introgressing any region from PT or Mi21 into B73 will cause diverse effects on gene expression, and that *Inv4m* is not exceptional in the magnitude of these perturbations. We expect that this is a common feature of inversion loci because of their size and high linkage disequilibrium relative to other classes of variants, and provides a challenge for functional studies to separate the important functional variation from the background linked variation.

We identified a set of genes within *Inv4m* that are worth pursuing as candidate causal genes for *Inv4m* function by combining our whole-transcriptome and *cis*-regulatory analyses. Due to the strong linkage across this locus, fine-mapping is unlikely to help refine this list. However, population genetic analyses of nucleotide diversity and divergence may be useful for prioritizing genes. Theoretical work suggests that fine-scale population-genetic data may be able to localize important loci inside inversions by scanning for regions of especially high divergence among haplotypes [20]. In old inversions, rare double-recombination events or gene conversions among haplotypes should eventually reduce the divergence at loci distant from both inversion breakpoints and any key adaptive variants within the locus [20]. Similarly, in young inversions, levels of divergence will remain low for many generations until background mutations accumulate by drift, and so any previously-diverged loci capture by the locus may remain visible for some time. These regions of elevation divergence among haplotypes might be detected by fine-scale estimates of genetic divergence from whole-genome sequencing data. However, this method has rarely been applied successfully (*e.g.,* [62–65]). Our analysis of *F*_*ST*_ and nucleotide diversity (Fig 1C and 1D) found two suggestive regions of elevated divergence inside *Inv4m*. However, sequencing data from more individuals with both haplotypes and a de-novo reference assembly of the minor (highland) haplotype would make this analysis much more powerful.

Together, these results imply that the *Inv4m* locus has many effects on maize development and physiology, and therefore its contribution to local adaptation is complex and not simply a change to a previously discovered major flowering or yield-related gene. The incorporation of ∼ 13Mb of the genome of *mexicana* likely brought with it a large number of functional variants that have both positive and negative effects on many molecular traits, most of which are not macroscopically visible, but may still impact performance in different conditions.

## Conclusions

This study provides a broad characterization of an adaptive chromosomal inversion found in domesticated maize from the Mexican highlands. Our results give insight into the genetic mechanisms underlying the adaptive value of *Inv4m*. GWAS results show that *Inv4m* is associated with faster flowering and higher yield in highland common gardens. The molecular roles of genes within the inversion are summarised by the phenotypic effects of *Inv4m*-regulated genes (Table 2) and enriched GO terms (Table 3), and the candidate gene set within *Inv4m* (S2 Table). Fine-mapping in this region is required to further dissect the functional role of loci within *Inv4m*, but will have additional challenges due to suppressed recombination between heterokaryotypes. Novel genomic technologies, such as a CRISPR/CAS system [66] that can reverse the orientation of the High *Inv4m* haplotype could be used to induce recombination across the newly collinear genomic regions, allowing the localization of specific effects of the different variants linked in this locus.

## Materials and methods

### Population genetics of *Inv4m*

We downloaded unimputed genotype-by-sequencing (GBS) data from 94,726 loci on chromosome 4 for 4,845 maize plants from the SeeD-maize GWAS panel [33, 67] and ran a principal components analysis on all positions within the *Inv4m* locus (between 168832447 and 182596678 in AGPv2 coordinates, [31]) with < 25% missing data and minor allele frequencies > 0.05. PC1 explained 22% of the genetic variation among these plants in this interval. Scores on PC1 neatly divided plants into three groups, representing the two homozygous classes at *Inv4m* and their heterozygotes. We cross-referenced plants with landrace passport data from Germinate 3, the CIMMYT Maize Germinate Database germinate.cimmyt.org/maize/ and extracted country of origin, latitude, longitude and elevation records. All but 7 of the plants containing the minor haplotype at *Inv4m* were from Mexico, so to study associations with elevation and to calculate other diversity statistics, we subsetted to only those plants collected in Mexico.

To calculate the association of *Inv4m* with elevation, we divided landraces into 100m bins, calculated the haplotype frequency of the minor haplotype in each bin, and fit a loess curve to the log-transformed haplotype frequencies, weighted by the number of landraces in each bin. Based on this analysis, we labeled the minor haplotype in Mexico “High”, and the major haplotype “Low”. We used the R function *HWExact* from the *HardyWeinberg* *R* package to test genotype counts against the Hardy-Weinberg expectation. We calculated diversity statistics separately for plants homozygous for the “High” or “Low” haplotypes at *Inv4m* across all of chromosome 4 in 500 marker windows using TASSEL 5 [68]. Because many more plants were homozygous for the “Low” haplotype, we randomly sampled 371 landraces to calculate the diversity statistics to make the sample sizes equal.

To calculate nucleotide diversity statistics, we downloaded published whole genome sequencing reads from 9 Mexican landraces mapped to the AGPv4 reference genome [36] from /iplant/home/lilepisorus/landrace_AGPv4. As described by those authors, one of the six sequenced landraces from Mexican Highlands (RIMMA0677) carries the lowland haplotype at *Inv4m* and so was excluded. We calculated *π* within each population and *F*_*ST*_ between the two population using the Analysis of Next Generation Sequencing Data (ANGSD) v0.931-2 [69–72] across an ≈ 130*Mb* region including *Inv4m*. We lifted over coordinates of the inversion breakpoints using the EnsemblPlants Assembly Converter Tool https://plants.ensembl.org/Zea_mays/Tools/AssemblyConverter to: (171771502,185951149). We ran ANGSD with the following options:

-GL1 -uniqueOnly 1 -remove_bads 1 -only_proper_pairs 1
  -trim 0 -C 50 -minMapQ 20 -minQ 20
  -doMaf 2 -doMajorMinor 4 -doSaf 1
  following the tutorial at http://www.popgen.dk/angsd/index.php/Thetas,Tajima,Neutrality_tests. We calculated average between-population pairwise nucleotide diversity following the R scripts available here: https://github.com/tvkent/Pi-Between assuming non-reference alleles matched between populations. All analyses used a 1Mb sliding window with a step-size of 100Kb.

### Association of *Inv4m* with agronomic traits

We re-analyzed phenotypic data from the F1 Association Mapping (FOAM) panel of Romero-Navarro *et al* [33] and Gates *et al* [37] to more fully characterize associations signatures of *Inv4m*. Full descriptions of this experiment and data access are described in those references. We downloaded BLUPs for each trait and line from Germinate 3, and kept lines with GBS genotype data from Mexico. We fit a similar model to the GWAS model used by Gates *et al* [37] to estimate the effect of *Inv4m* genotype on the trait’s intercept and slope on trial elevation, accounting for effects of tester ID in each field and genetic background and family effects on the trait intercept and slope using four independent random effects. We implemented this model in the *R* package *GridLMM* [73]. We extracted effect sizes and covariances conditional on the REML variance component estimates and used these to calculate standard errors for the total *Inv4m* effect as a function of elevation. To test whether the phenotypic effects of *Inv4m* on yield components could be explained as indirect effects via flowering time, we additionally re-fit each model using Days-To-Anthesis as a covariate with an independent effect in each trial.

### Experimental material for isolating *Inv4m*

To directly assess phenotypic effects of the *Inv4m* locus, we selected two highland landrace accessions which both carry the High haplotype of *Inv4m*, Palomero Toluqueno (PT) and an accession from the Cónico landrace. These *Inv4m* landrace accessions were obtained through the International Maize and Wheat Improvement Center (CIMMYT); PT came from accession mexi5 (referred to henceforth as PT) and Cónico from accession Michoacán 21 (referred to henceforth as Mi21). B73 is a modern inbred from the United States that carries the non-inverted *Inv4m* haplotype. Both landraces were crossed with B73 and one resulting F1 individual from each cross was backcrossed to B73 for 5 generations, selecting on a diagnostic SNP for *Inv4m* each cycle with a cleaved amplified polymorphic sequence (CAPS) assay. The diagnostic SNP is at position 4:179617762 (Matt Hufford, Personal communication). DNA was extracted from leaf tissue using a Urea lysis buffer extraction protocol (https://github.com/RILAB/lab-docs/wiki/Wetlab-Protocols). Primers were designed to amplify the fragment of DNA carrying the diagnostic SNP (Forward: CTGAGCAGGAGATGATGGCCACTC; Reverse: GGAAAGGACATAAAAGAAAGGTGCA). Amplification consisted of 5 minute denaturation at 95°C, 35 cycles of 95-60-72°C for 30 seconds each, 7 minutes of final extension step at 72°C, followed by a 4°C hold. Amplified DNA was then digested with the *Hinf 1* enzyme for 1 hour at 37°C, and the resulting product was run out on a 1% agarose gel for genotyping. Two of the resulting BC_5_ individuals per population identified as heterozygous for *Inv4m* were self-pollinated to produce BC_5_S_1_ families segregating for *Inv4m*.

### Growth chamber experiment to identify phenotypic effects of *Inv4m*

We planted seeds from the four segregating families (two parents per *Inv4m* donor) in the UC Davis controlled environment facility growth chambers. Chambers were programmed to mimic temperatures in Mexican lowlands (22°C night, 32°C day, 12 hr light) and highlands (11°C night, 22°C day, 12 hr light). Kernels were soaked in distilled H_2_0 for 12 hours and planted in 10.2cm x 34.3cm nursery pots (Steuwe & Sons: CP413CH) in a substrate mixture composed of a 3:1 ratio of Sungro Sunshine Mix #1 to sand. Pots were organized in racks with 9 pots per rack (Steuwe & Sons: tray10). Plants were watered every other day with a 1x Hoagland nutrient solution, and emergence was recorded daily. The experiment was replicated and growth chambers were switched to account for variation between instruments between replicates (See S1 Fig for a graphical workflow). The first replicate of the experiment began March, 2017 and the second replicate began April, 2017.

Two seeds were planted in each pot, one in the center and one near the corner, and a total of nine tissues were sampled from the two plants when they reached specific developmental stages. These nine tissues were selected to maximize the diversity of gene expression profiles based on the transcription atlas of [39]. Corner plants were removed from the pot and sampled when they reached the V1 stage, while center plants were sampled when they reached the V3 stage (S2 Fig). Two tissue types were sampled from the V1 stage, and 7 tissue types were sampled from the V3 stage (Table 1). Sampling occurred between 2 and 4 hours after simulated sunrise. Plant tissue was placed in 2 ml centrifuge tubes, immediately flash frozen in liquid nitrogen, and stored at -70°C.

### *Inv4m* Genotyping and RNA sequencing

We used the same DNA extraction and CAPS genotyping methods as previously described to genotype the seedlings for the *Inv4m* haplotype. We harvested and successfully genotyped 364 individual plants from families segregating for *Inv4m*. The Low, Heterozygote, and High *Inv4m* haplotypes were segregating in the PT families at a 42:78:45 ratio, within Hardy-Weinberg equilibrium (HWE; D = -2.24, p-value = 0.53). The inversion was segregating in the Mi21 families at a 59:97:43 ratio, also within HWE (D = -0.928392, p-value = 0.7768964).

We randomly sampled 3 biological replicates per experimental replicated from each tissue and temperature treatment for the two homozygous *Inv4m* genotypes per donor, for a total of 432 samples from 96 plants. Approximately 20 mg of tissue from each sample was placed in a 2ml centrifuge tube, flash-frozen in liquid nitrogen and ground using stainless steel beads in a SPEX Geno/Grinder (Metuchen, NJ, USA). mRNA was extracted using oligo (dT)_25_ beads (DYNABEADS direct) to isolate polyadenylated mRNA using the double-elution protocol. We prepared randomly primed, strand specific, mRNA-seq libraries using the BRaD-seq [74] protocol with 14 PCR cycles. Samples underwent a single carboxyl bead clean-up, were quantified using the Quant-iT PicoGreen dsDNA kit, and then normalized. We took 2ng per library and multiplexed 96 samples for sequencing. Each multiplexed library was sequenced on 1 lane of a Illumina HiSeq X platform, generating a mean of 4,241,500 reads per sample. Raw reads were quality checked using FastQC v.0.11.5 [75]. Adapter sequences, low quality reads (q<20), and sequences less than 25 bp were removed using Trimmomatic v.0.36 [76].

### Effects of genotype at *Inv4m* on seedling emergence

The effect of *Inv4m* on seedling emergence were analyzed using the following random slope and intercept model for each donor and temperature treatment separately:

$$\begin{array}{c} y_{ijk}∼\mu +\beta_{1}G_{i}+u_{ijk}+e_{ijk} \end{array}$$ (Mod 1)

*y*_*ijk*_ is the emergence time for individual plant *k* in experimental replicate *j* in *Inv4m* genotype *i*. *μ* is the model intercept, *β*_1_*G*_*i*_ is the effect of *Inv4m* genotype, **u** = **u_ijk_** is a random effects term for the experimental replicate, and *e*_*ijkl*_ is residual error. Variance components, coefficients and standard errors were estimated by REML using the *R* function *lmer* [77], and p-values were calculated using conditional F-tests [78].

### Population characterization

BC_5_S_1_ plants are expected to contain ∼ 3% residual DNA inherited from the donor parent across the remaining 9 chromosomes. We used the RNAseq reads from each plant to genotype all residual regions across the genome by calling variants in the expressed regions. Paired reads that passed filtering were aligned to the B73 reference genome version 4 [79] using hisat2 [80], and variant loci were called using GATKv3 [81, 82]. We ran MarkDuplicates, SplitNCigarReads and HaplotypeCaller on every sample, including all 435 samples from our segregating families and an additional 46 B73, 48 PT and 47 B73-PT F1 samples from plants run in parallel with the families but were not otherwise used in this experiment, and then ran GenotypeGVCFs on all samples jointly. We next used SelectVariants to extract SNPs, and VariantFiltration to remove SNPs with FS score < 30 and QD > 2.0. We further filtered for SNPs called homozygous-reference in all B73 samples, and which exhibited allele frequencies > 1/8 and < 7/8 in either the PT or Mi21 families (expected frequencies of each variant should be 0.25 or 0.5 depending on recombination between the two BC_5_ parents of each population, but we allowed for some sampling error). We used this set of highly filtered SNPs for each population to genotype each of the individual plants. For each plant, at each locus we first combined all genotype likelihoods across all RNA samples from the same sample. We then identified the approximate breakpoints of the introgressed regions by inspecting the density of variant sites. We identified 3 regions (on chromosomes 2, 4 and 5) in the PT family and 2 regions in the Mi21 family (on chromosomes 3 and 4). Within these introgressed regions, we used R/QTL [83] to assign genotype probabilities across the *Inv4m* locus for each plant, allowing error.prob = 0.2. Finally, we observed that several genes outside these 5 introgressed regions each of which exhibited >= 2 SNPs relative to the reference. We hypothesized that these genes may have a different chromosome location in the landraces relative to B73, and actually reside inside one of the 5 introgressed regions. We therefore assigned their genotype to the most common genotype among these variant loci.

### RNA quantification

To quantify gene expression, we ran kallisto v.0.42.3 [84] separately on each sample using the B73 AGPv4.36 transcript models downloaded from the maize genome database [79]. We limited to only one bootstrap replicate, and then summed the transcript counts for each gene. Genes were retained for analysis when at least a third of the samples had 10 or more reads. Gene counts were normalized using the weighted trimmed mean of M-values (TMM) with the *calcNormFactors* function in *edgeR* [85]. Normalization using TMM reduces bias of very highly and lowly expressed genes. The *voom* function [86] in the *limma* package [87] was used to convert normalized reads to log2-counts per million (log2CPM), estimate a mean-variance relationship, and assign each observation a weight based on its predicted variance. Observation-weights were used in downstream analyses to account for heteroscedasticity. We estimated batch effects using the *removeBatchEffects* function in limma using the experimental replicate as batch, which corrected the log2CPM expression values. Global patterns of gene expression across the experiment were visualized with the *plotMDS* function from *edgeR*.

### Analysis of *Inv4m* effects on gene expression

We divided genes into three groups to estimate the effects of *Inv4m* or other introgressed landrace alleles, based on whether each gene resided in a “clean” genomic region with only B73’s allele present in the families, inside the *Inv4m* locus itself, or if it resided within one of the genomic blocks containing any of the residual landrace genome outside of *Inv4m*. Each group of genes served a different purpose in the analysis of *Inv4m*. Genes in the “clean” region were used to assess the effects of *Inv4m* on global gene expression and indirectly assess the effects on development and physiology more broadly. Genes inside the *Inv4m* locus were scanned for candidate alleles underlying *Inv4m’s* effects. Genes in the residual introgression blocks were used as controls to assess the similarity of PT and Mi21 alleles in random genomic loci, as well as compare effect sizes and expression correlations with *Inv4m*.

For genes that resided in “clean” genomic regions with only B73’s allele present in the segregating families (approximately 89.8% of genes expressed in both donors), we estimated the effect of the *Inv4m* locus separately in each *Inv4m* donor, temperature treatment, and tissue using the linear model:

$$\begin{array}{c} y_{ij}=\mu +\beta {\text{Inv4m}}_{i}+e_{ij}\;e_{ij}∼\text{N}\left(0,\phi_{ij}\sigma^{2}\right) \end{array}$$ (Mod 2)

where *y*_*ij*_ is a normalized, batch-corrected log2CPM value for a single gene in a single sample, *μ* is the intercept for that gene in the particular population, environment and tissue, *β* is the corresponding effect of the landrace *Inv4m* haplotype, and *e*_*ij*_ is the model residual, which is assumed to be independent of all other residuals and have variance proportion to *ϕ*_*ij*_, the empirical weight factor calculated by voom. We fit this model to the whole set of “clean” genes using the *lmFit* function from *limma*, and extracted *β* and its standard error (|*β*|/*t*). This provided 18 independent *Inv4m* effect-size estimates and standard errors for each gene per family. Since our goal was to maximize our power to identify *any* effect of *Inv4m*, we combined results across conditions using the *mash* method [42] implemented in the *mashr* R package. *mash* leverages correlations across conditions to more accurately estimate effect size and variance statistics. *mashr* requires complete data (*i.e.* each gene requires an effect size estimate and standard error in every condition). Since some genes in particular tissue:temperature conditions were excluded above due to low expression, we imputed their effect sizes as 0 with standard error set to 1000 to convey our lack of knowledge about the true effect size of *Inv4m* in these conditions. We used the output of *mashr* to identify a union set of genes regulated by *Inv4m* by identifying genes with *lfsr* < 0.05 in any condition.

We also fit a separate model to test for interactions between *Inv4m* and the temperature environment, separately for each *Inv4m* donor and tissue:

$$\begin{array}{c} y_{ijk}=\mu +\beta_{1}{\text{Inv4m}}_{i}+\beta_{2}{\text{Temp}}_{j}+\beta_{3}{\text{Inv4m:Temp}}_{ij}+e_{ijk}\;e_{ijk}∼\text{N}\left(0,\phi_{ij}\sigma^{2}\right) \end{array}$$ (Mod 3)

This model adds *β*_2_, the main effect of temperature on expression, and *β*_3_, the interaction between *Inv4m* and temperature. However it is less flexible than the first model because the residual variance *σ*^2^ is constrained to be equal for the two temperature environments. This model was also fit to each gene using *lmFit*, and the estimate of *β*_3_ and its standard error were extracted. We again used *mashr* to identify the union set of genes affected by this interaction.

For genes residing inside the *Inv4m* locus, we fit the same two statistical models with the *lmFit* function. However, we did not include these genes in the multiple adaptive shrinkage analysis.

For genes residing outside *Inv4m*, but within one of the genomic blocks containing residual landrace DNA in both donors, we fit a slightly different statistical model:

$$\begin{array}{c} y_{ij}=\mu +\beta {\text{cis}}_{i}+e_{ij}\;e_{ij}∼\text{N}\left(0,\phi_{ij}\sigma^{2}\right) \end{array}$$ (Mod 4)

where *cis* is the local genotype of the gene, and *β* is the associated effect. For genes in residual genomic blocks on chromosome 4, the *cis* genotypes were highly correlated with the *Inv4m*, so some of the *cis* effect may have been caused by *Inv4m*, but these effects were difficult to separate statistically. However for genes on other chromosomes, the two genotypes were largely uncorrelated.

### Sequence divergence and expression correlation between donors within the High *Inv4m* haplotype and the residual genomic regions

We estimated the sequence divergence between the two landrace donors and B73 for each gene within the chromosome 4 introgression containing *Inv4m* present in both families. For each gene, we calculated the genetic similarity of the PT and Mi21 alleles relative to B73 by counting the number of shared SNPs divided by total number of observed SNPs within each gene window, using only the highly filtered SNP set described above. We calculated the correlation in expression effect sizes due to *Inv4m* between PT and Mi21 for each tissue and temperature combination. T-tests were used to determine whether genetic similarity and expression correlation were higher within *Inv4m* relative to the shared introgressed region.

### Gene ontology enrichment

Genes were assigned to gene ontology (GO) categories for functional annotation using an updated ontology annotation [88] which we expanded to include all ancestral terms for each gene with the *buildGOmap* function of the R package clusterProfiler [89]. Genes in “clean” genomic regions which responded to the High *Inv4m* haplotype in the same direction in both donor populations were classified as *Inv4m-regulated* and tested as foreground genes in a GO enrichment analysis. Genes that were expressed in each tissue and temperature combination in both donor populations, but were not *Inv4m-regulated* were included in the set of background genes. We calculated the enrichment of each GO term using the *enricher* function in the clusterProfiler R package. We selected GO terms with a false discovery rate less than 1% after a Benjamini-Hochberg multiple test correction. We then calculated the percent of genes in each GO terms that were *Inv4m-regulated*, and ranked all GO terms by their maximum enrichment across conditions. We then selected the highest enriched GO term among each set of GO terms that had a semantic-similarity >0.5 as the representative term set.

### Candidate gene pathway assessment among *Inv4m-regulated* genes

We inspected two additional candidate gene sets: genes known to regulate flowering time in maize from two separate sources [45, 46], and genes regulated by the microRNA miR172. *Inv4m* has previously been associated with flowering [33]. miR172 is a highly conserved micro-RNA across the plant kingdom that regulates development and flowering time and one miR172 gene, *miR172c* is located inside *Inv4m*. For miR172 targets, we found the mature sequence for zma-miR172c: “AGAAUCUUGAUGAUGCUGCA” from miRBase http://www.mirbase.org, and used this as a query of the Plant Small RNA Target Analysis Server (psRNATarget, http://plantgrn.noble.org/psRNATarget), and collected all predicted target genes. We also used TAPIR’s pre-computed target genes for *zma-miR172a-b-c-d*. These two categories of genes were inspected by hand for evidence of regulation by *Inv4m*. From each of the candidate lists, we reported genes which were in the clean genetic background, and differentially expressed in a majority of tissues in the same direction.

### Candidate genes within *Inv4m*

We used the intersection of two separate methods to identify candidate adaptive genes within *Inv4m*. First, we quantified the proportion of conditions (tissue:temperature combinations) that each gene within *Inv4m* was differentially expressed according to *Inv4m* genotype. To be considered differentially expressed, the gene needed to be differentially expressed in both *Inv4m* donor populations and in the same direction. Genes where at least one donor was not expressed were removed from this analysis per condition. As a complimentary approach, we calculated the correlation in expression between each *Inv4m* gene and all the genes in “clean” genomic regions that we determined to be *Inv4m-regulated* above. We then quantified the proportion of these correlations that were significant and in the same direction in both donor populations for each gene. For this analysis, we used the *lm* function in R to implement a linear model with the *Inv4m-regulated* gene expression as the response variable, and the *Inv4m* gene’s expression and *Inv4m* genotype as predictors.

### De-novo assembly of novel genes

We collected all un-mapped reads from the samples homozygous for the high *Inv4m* haplotype, and used Trinity v2.4 [90] to assemble un-annotated transcripts using default settings. We then used Kallisto to quantify the expression of each of these novel transcripts using the un-mapped reads from each RNAseq sample. To search for candidate “novel” genes in the highland haplotype, we filtered for Trinity genes that had zero estimated counts in any of the samples that were homozygous for the B73 haplotype of *Inv4m*, but had non-zero estimated counts in at least 2 samples homozygous for the PT or Mi21 haplotype in each segregating donor family (to exclude genes that may reside in either PT or Mi21).

## Supporting information

S1 Fig Graphical workflow of gene expression analytical pipeline. (PDF)

S2 Fig Photographs of how shoot tissues were sampled from plants at the V3 stage. (PDF)

S3 Fig Multidimensional scaling plot of normalized and batch corrected gene expression (log2CPM) of the 500 most variable genes across our dataset using the *plotMDS* function. The plot represents a two-dimensional log2 fold change distance between each sample colored in three different ways to display how each factor effects the structure of the data. A) tissue B) temperature and C) *Inv4m* genotype.
(PDF)

S4 Fig Correlation between local genotype and genotype at *Inv4m* of any gene with a landrace allele in each family. Vertical line represents the location of *Inv4m*.
(PDF)

S5 Fig Standardized similarity indices of genetic and expression calculated on a per gene basis between PT and Mi21. Vertical lines represent the boundaries of *Inv4m*. The landraces are more similar within *Inv4m* for both metrics.
(PDF)

S6 Fig Boxplots are effect sizes of *Inv4m* in each segregating landrace family across 6 tissues in each temperature treatment. Panel A) are nuclear genes in the chloroplast thylakoid gene ontology category, GO:0009534. Notice that genes in the S2 leaf base in the warm are downregulated. Panel B) are effect sizes of *Inv4m* on chloroplastic genes in the chloroplast genome.
(PDF)

S1 Table Summary of linear mixed effects model of seedling emergence for each *Inv4m* donor and temperature treatment. (PNG)

S2 Table Candidate genes within the High *Inv4m* haplotype. The List column identifies how genes got on the list from the original list of 89. Correlated trans means that they are correlated with more than 3% of genes that were *Inv4m-regulated*, consistent DE are genes that were differentially expressed in more than 90% of the tissue:temperature conditions that they were expressed in, and TF = transcription factor.
(PNG)

S3 Table Validated maize flowering genes from two sources (Dong et. al. 2012, Tenaillon et. al. 2018) that were Inv4m-regulated in the same direction in both landrace families. The tissue column represents the tissue and temperature treatment that the gene was differentially expressed in.
(PNG)

S4 Table miRNA172 target genes that were *Inv4m-regulated*. Homologous gene descriptions are from Arabidopsis thaliana database (https://www.arabidopsis.org/). GO annotation descriptions are the biological function (http://maizemine.rnet.missouri.edu/). TF = Transcription factor.
(PNG)
