# Supplementary figures and images for: Gene regulatory effects of a large chromosomal inversion in highland maize
Source: PLoS Genet. 2020 Dec 3;16(12):e1009213. doi: 10.1371/journal.pgen.1009213 (PMC7752097; doi:10.1371/journal.pgen.1009213)

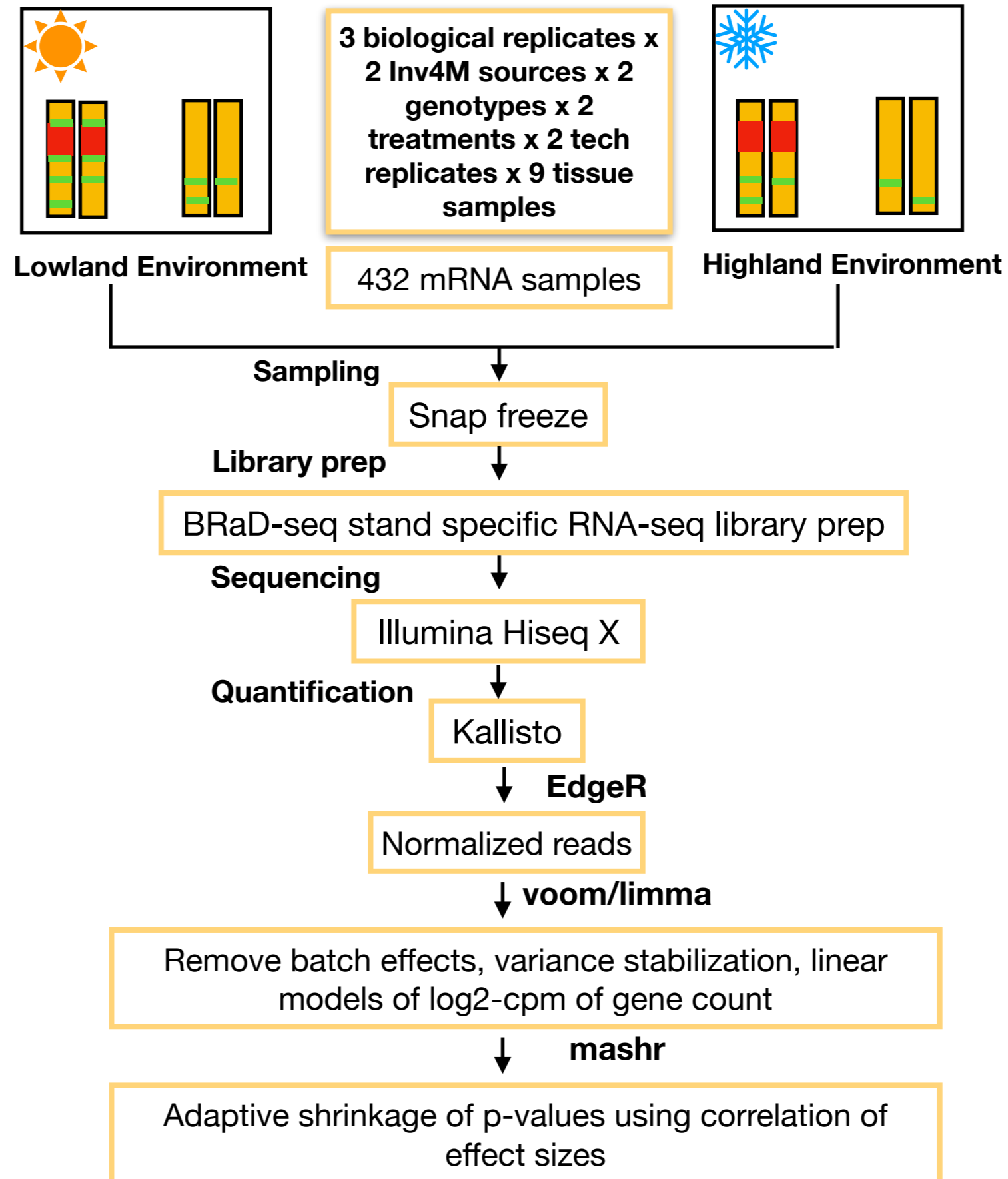

Supplement: S1 Fig — (PDF) [file pgen.1009213.s001.pdf]

A)

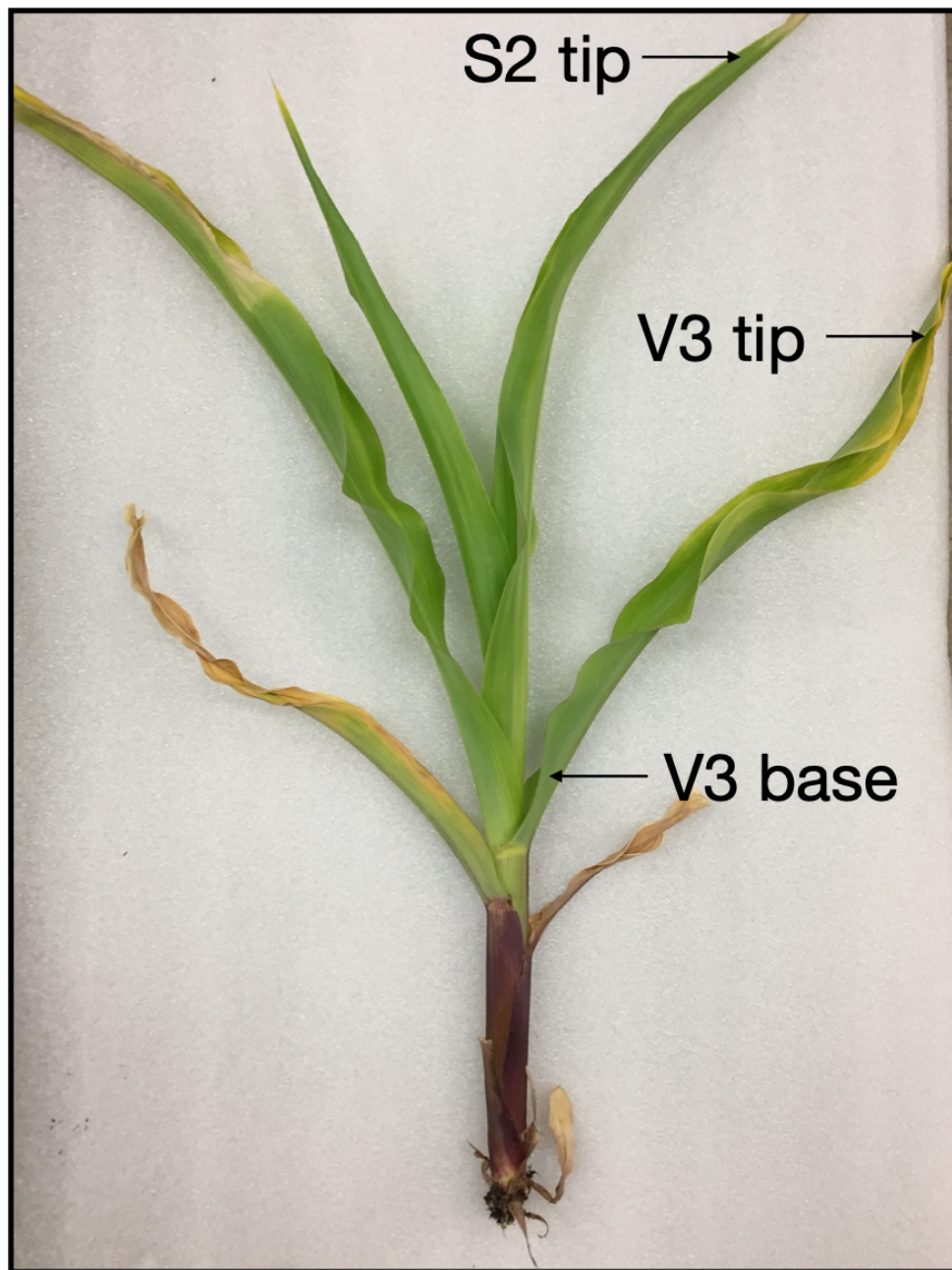

B)

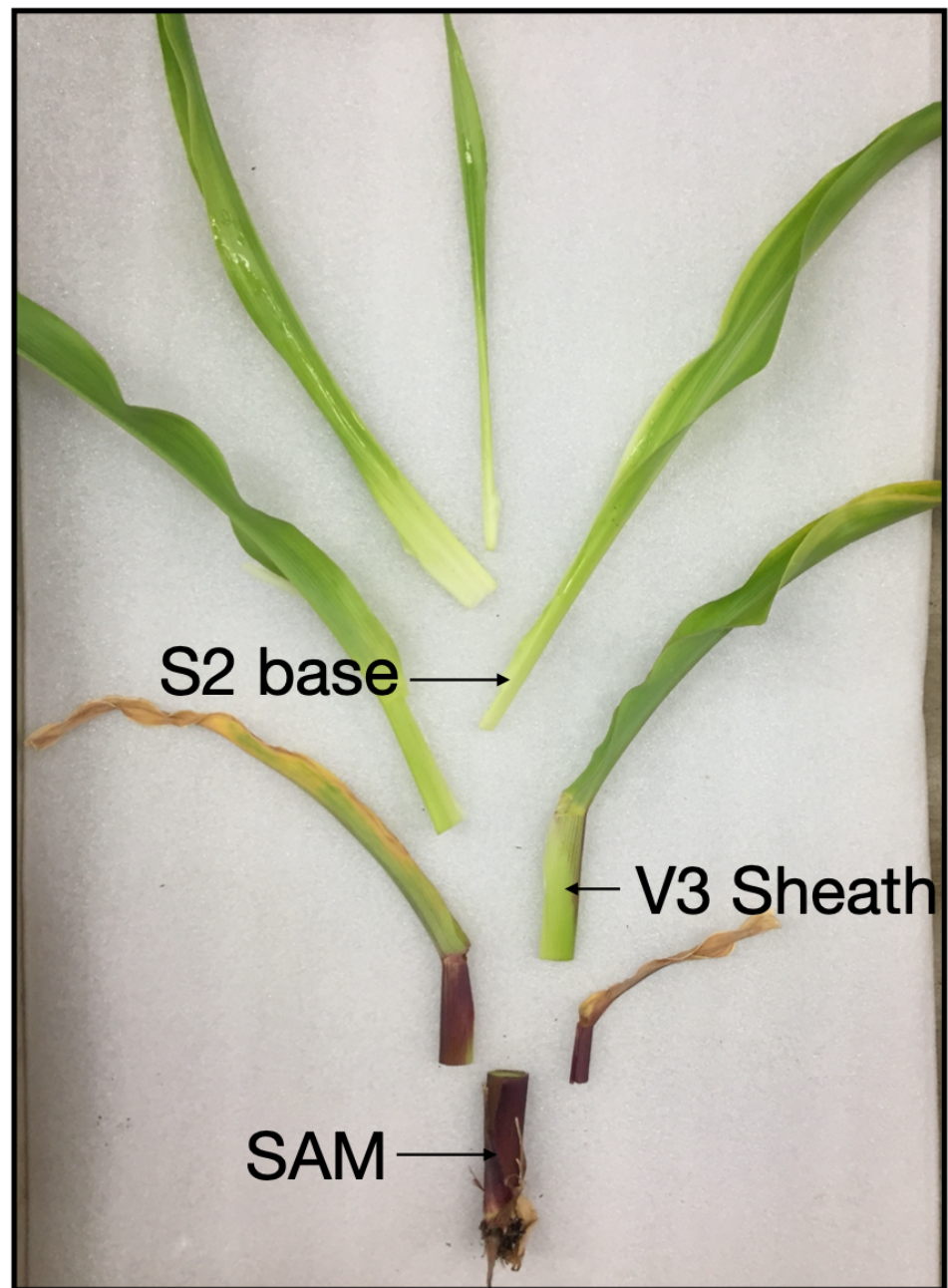

Supplement: S2 Fig — (PDF) [file pgen.1009213.s002.pdf]

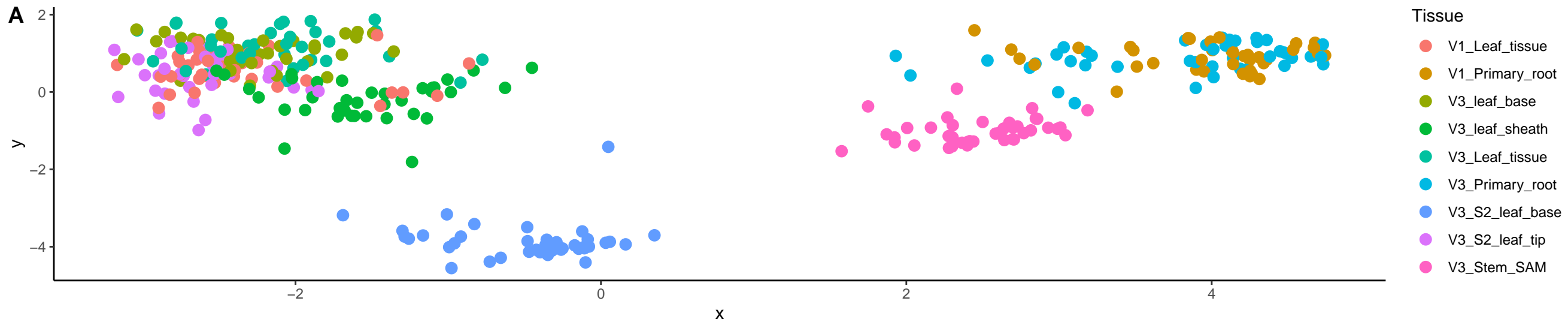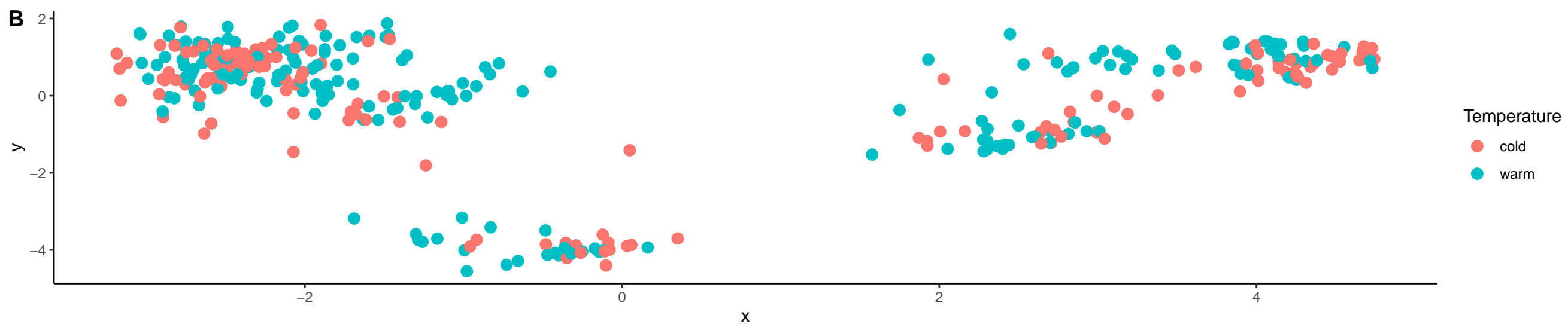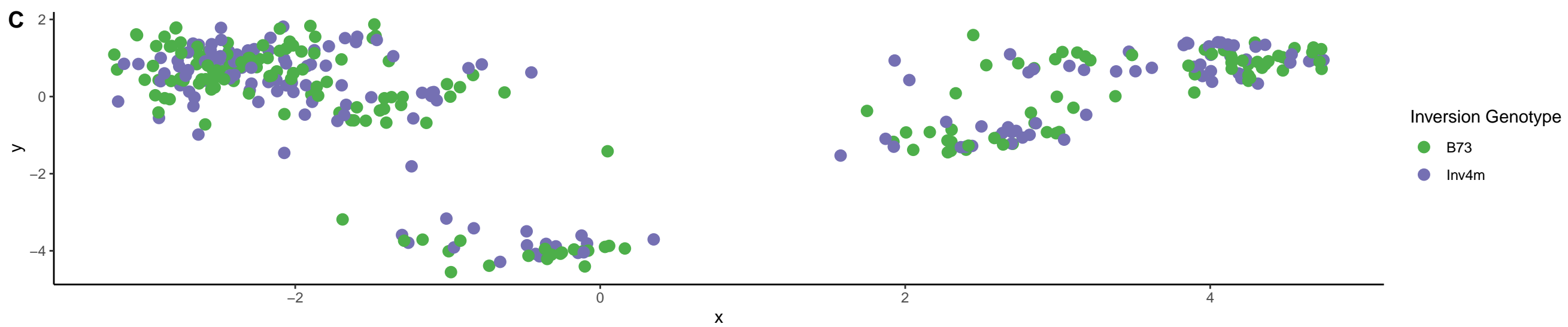

Supplement: S3 Fig — The plot represents a two-dimensional log2 fold change distance between each sample colored in three different ways to display how each factor effects the structure of the data. A) tissue B) temperature and C) Inv4m genotype. (PDF) [file pgen.1009213.s003.pdf]

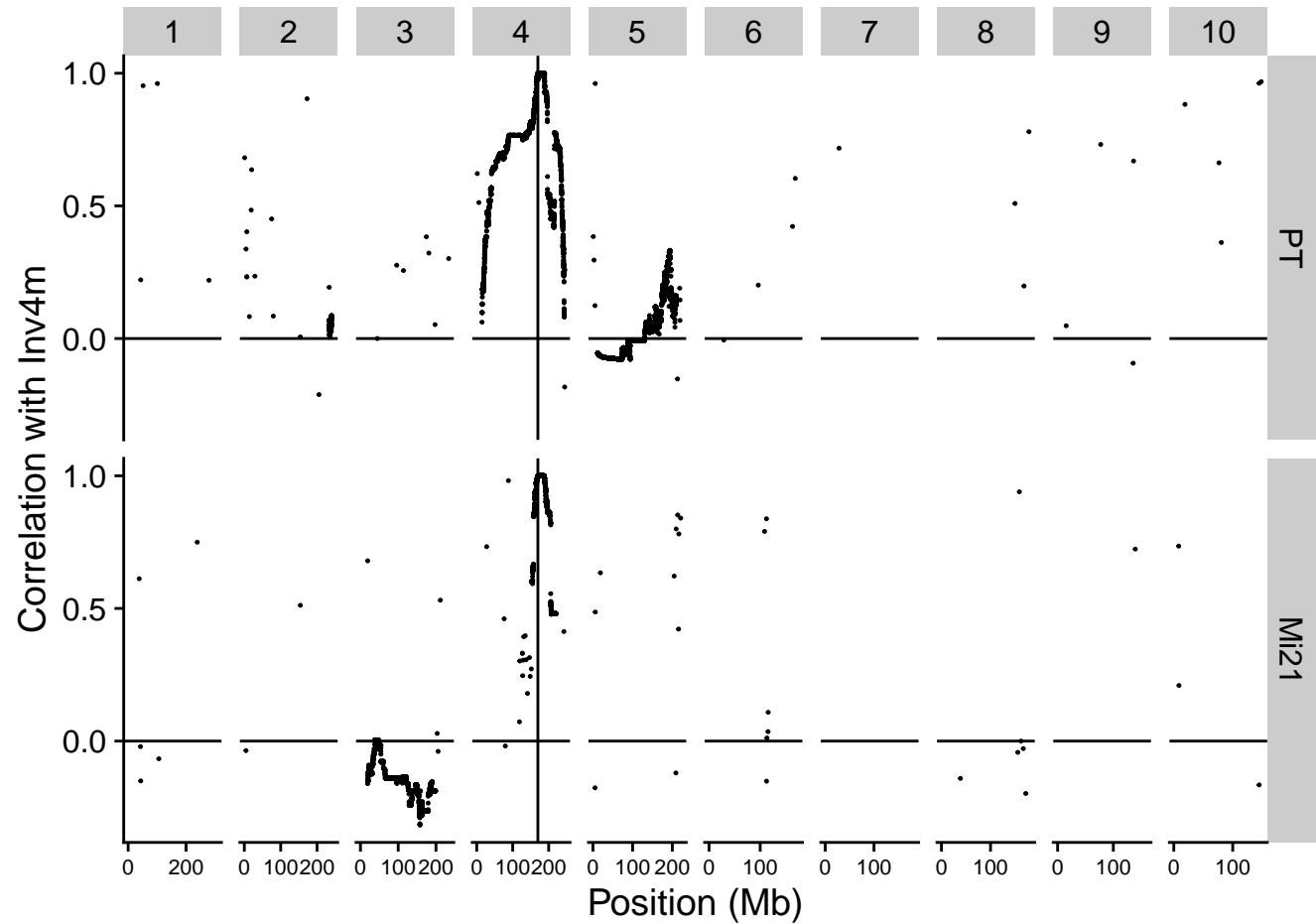

Supplement: S4 Fig — Vertical line represents the location of Inv4m. (PDF) [file pgen.1009213.s004.pdf]

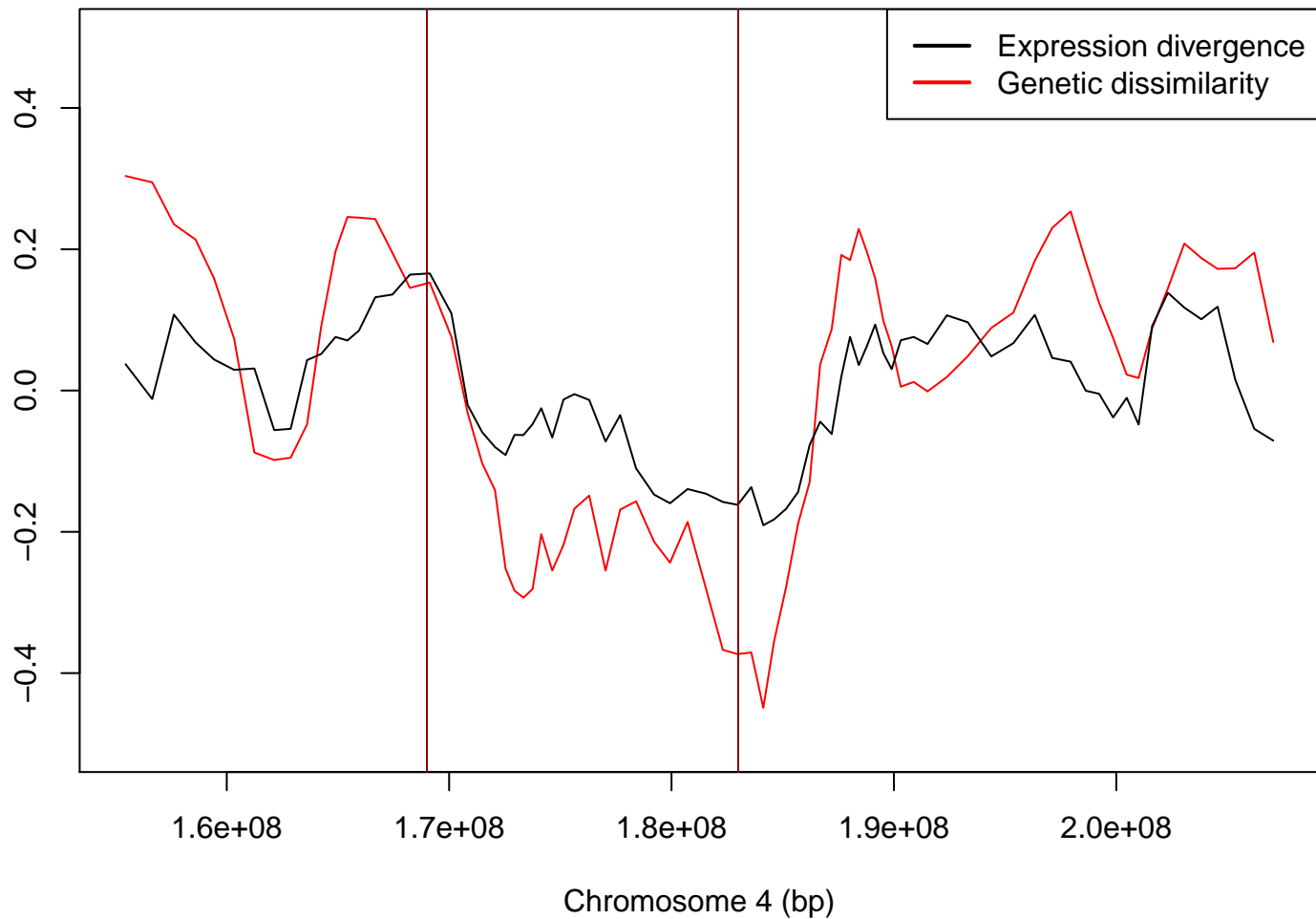

Supplement: S5 Fig — Vertical lines represent the boundaries of Inv4m. The landraces are more similar within Inv4m for both metrics. (PDF) [file pgen.1009213.s005.pdf]

GO:Chloroplast thylakoid

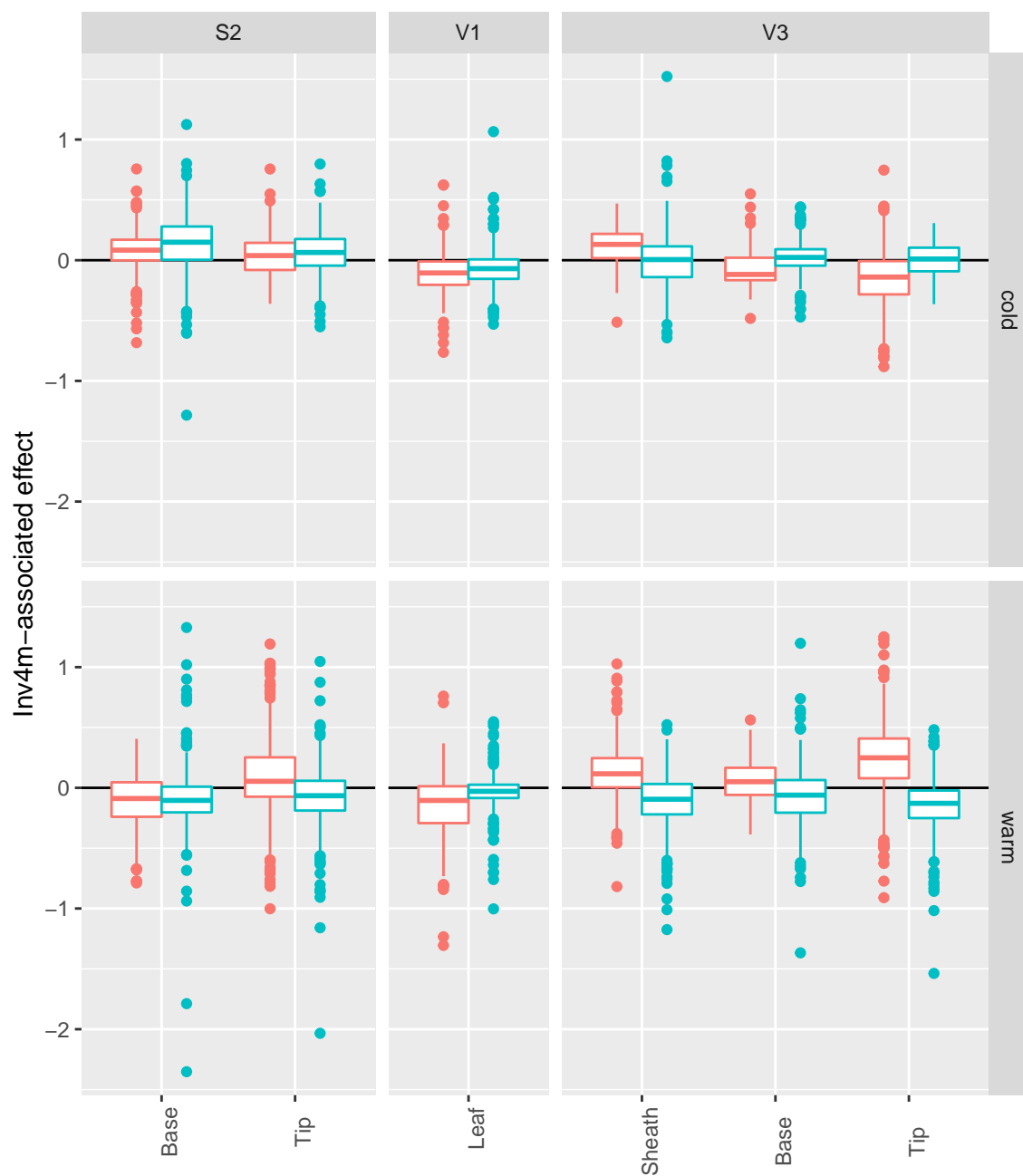

Chloroplast genome

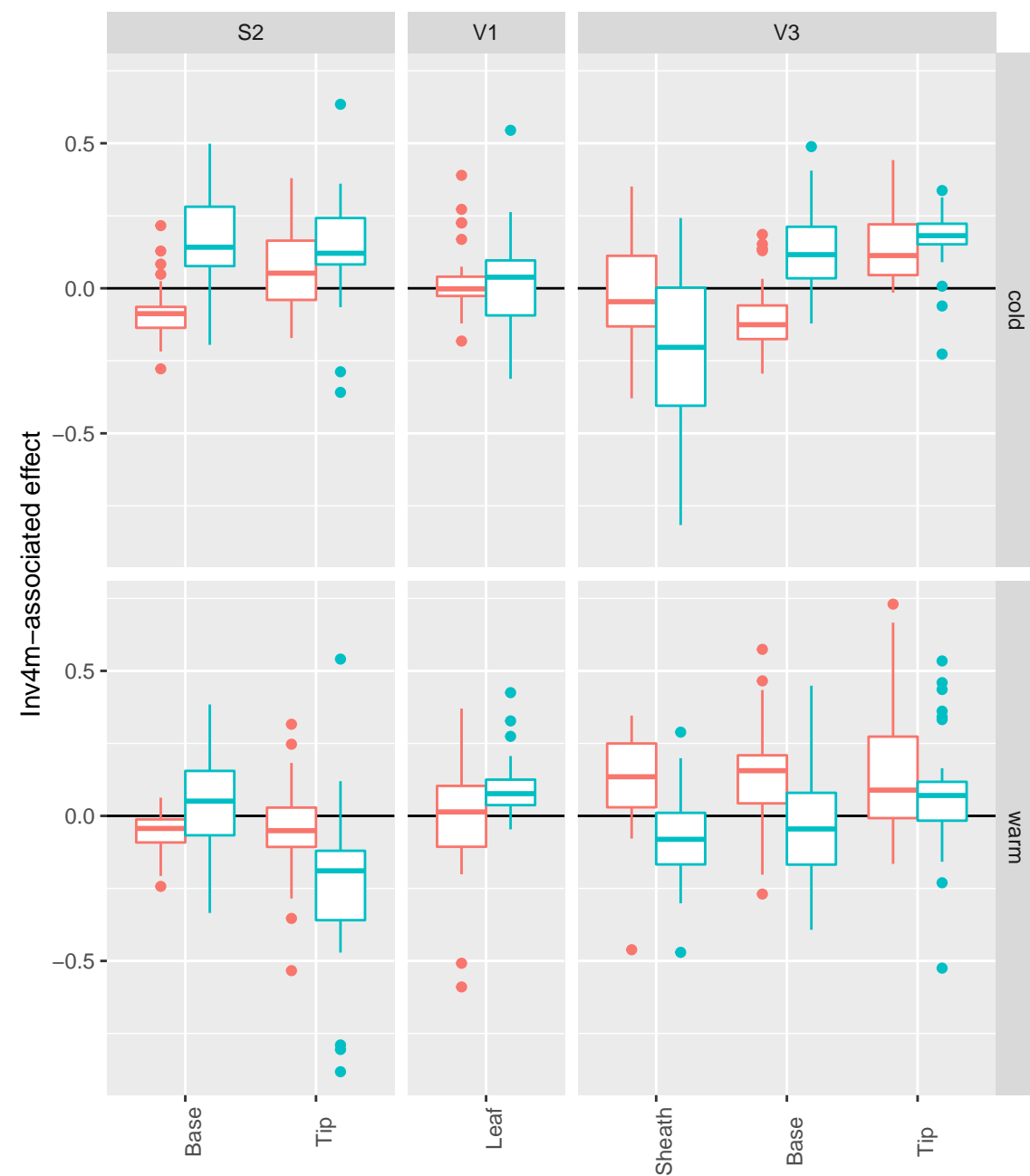

Pop PT Mi21

Supplement: S6 Fig — Panel A) are nuclear genes in the chloroplast thylakoid gene ontology category, GO:0009534. Notice that genes in the S2 leaf base in the warm are downregulated. Panel B) are effect sizes of Inv4m on chloroplastic genes in the chloroplast genome. (PDF) [file pgen.1009213.s006.pdf]

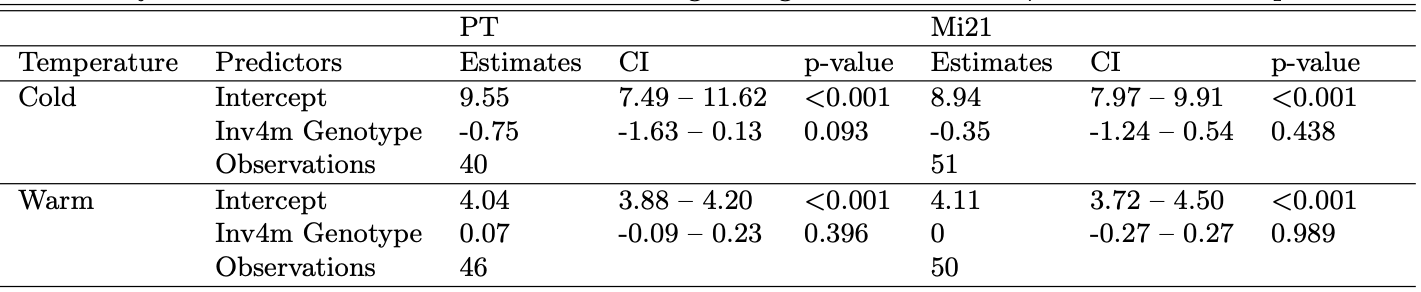

Supplement: S1 Table — (PNG) [file pgen.1009213.s007.png]

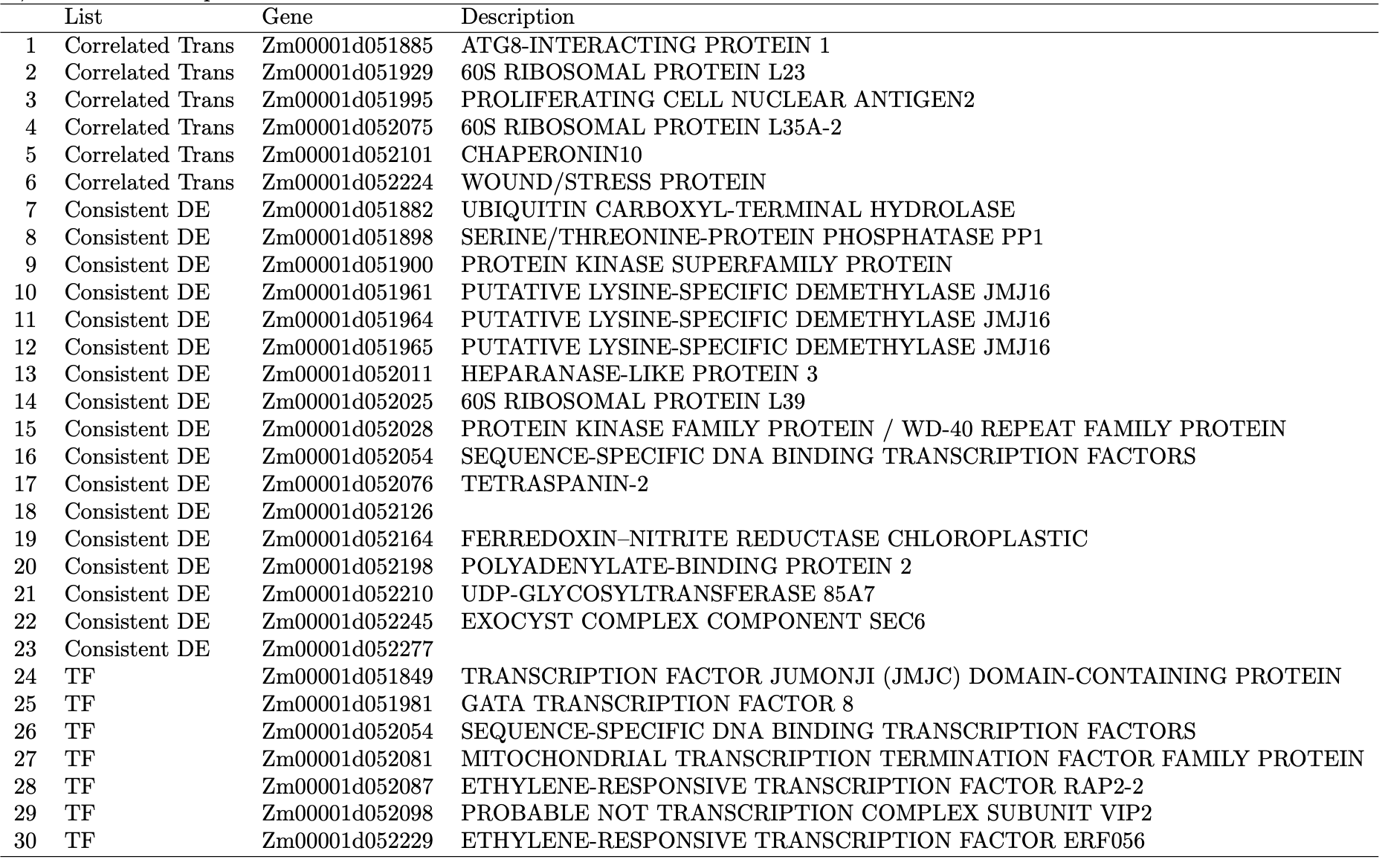

Supplement: S2 Table — The List column identifies how genes got on the list from the original list of 89. Correlated trans means that they are correlated with more than 3% of genes that were Inv4m-regulated, consistent DE are genes that were differentially expressed in more than 90% of the tissue:temperature conditions that they were expressed in, and TF = transcription factor. (PNG) [file pgen.1009213.s008.png]

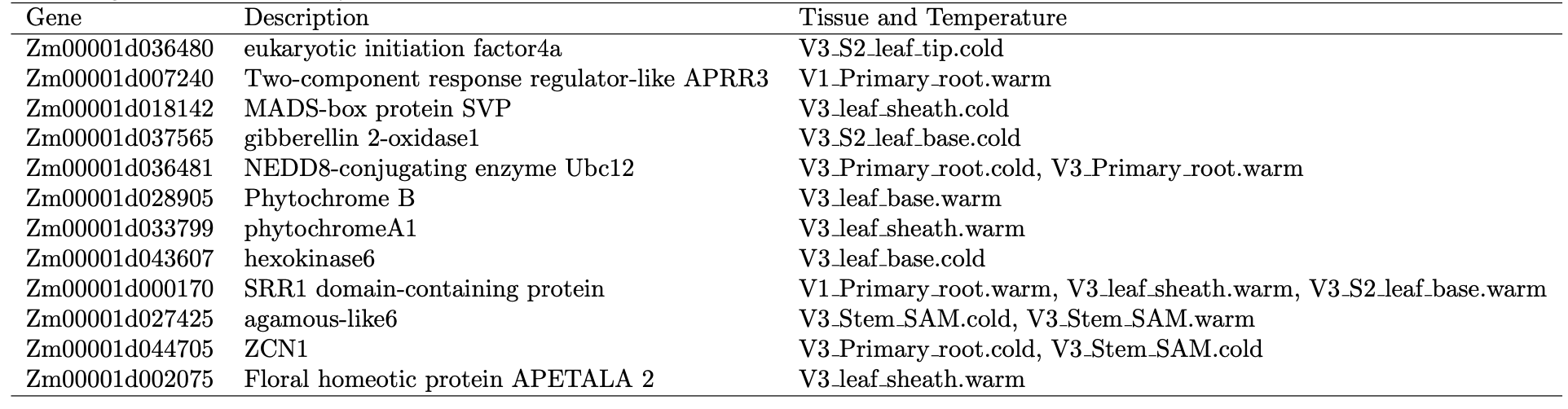

Supplement: S3 Table — The tissue column represents the tissue and temperature treatment that the gene was differentially expressed in. (PNG) [file pgen.1009213.s009.png]

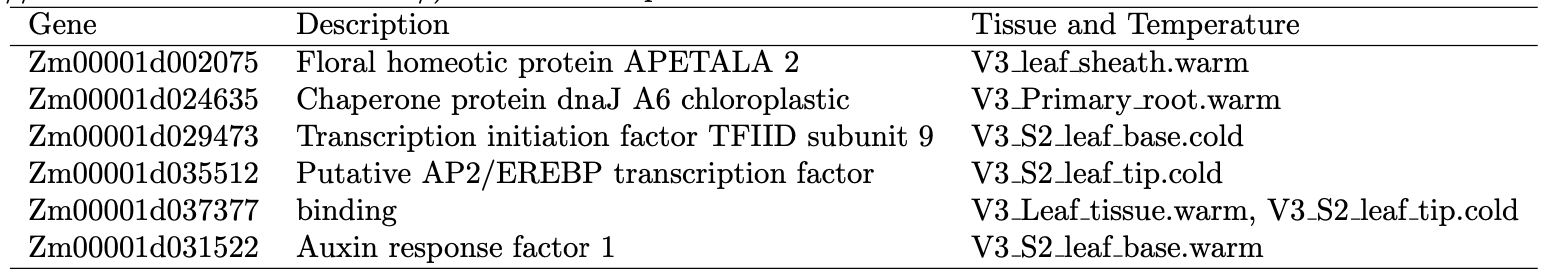

Supplement: S4 Table — Homologous gene descriptions are from Arabidopsis thaliana database (https://www.arabidopsis.org/). GO annotation descriptions are the biological function (http://maizemine.rnet.missouri.edu/). TF = Transcription factor. (PNG) [file pgen.1009213.s010.png]
